# *Plasmodium knowlesi* tryptophan-rich antigen 40.1 binds human erythrocyte spectrin alpha

**DOI:** 10.1371/journal.pntd.0014700

**Published:** 2026-09-17

**Authors:** Wang-Jong Lee, Fadhila Fitriana, Jadidan Hada Syahada, Johnsy Mary Louis, Hyun-Young Sim, Ernest Mazigo, Hojong Jun, Fauzi Muh, Feng Lu, Seok Ho Cha, Se Jin Lee, Sunghun Na, Wanjoo Chun, Won Sun Park, Yee Ling Lau, Robert W. Moon, Eun-Taek Han, Jin-Hee Han

**Affiliations:** 1 Department of Tropical Medicine, School of Medicine, Kangwon National University, Chuncheon, Republic of Korea; 2 Department of Epidemiology and Tropical Diseases, Faculty of Public Health, Universitas Diponegoro, Semarang, Indonesia; 3 Department of Parasitic Diseases and Vector Control, National Institute for Medical Research, Dar es Salaam, Tanzania; 4 Department of Pathogen Biology and Immunology, School of Medicine, Yangzhou University, Yangzhou, China; 5 Department of Parasitology and Tropical Medicine, Inha University School of Medicine, Incheon, Republic of Korea; 6 Department of Obstetrics and Gynecology, Kangwon National University Hospital, Chuncheon, Republic of Korea; 7 Department of Pharmacology, School of Medicine, Kangwon National University, Chuncheon, Republic of Korea; 8 Department of Physiology, School of Medicine, Kangwon National University, Chuncheon, Republic of Korea; 9 Department of Parasitology, Faculty of Medicine, Universiti Malaya, Kuala Lumpur, Malaysia; 10 Department of Infection Biology, Faculty of Infectious and Tropical Diseases, London School of Hygiene and Tropical Medicine, London, United Kingdom; 11 Institute of Medical Sciences, Kangwon National University, Chuncheon, Republic of Korea; National University of Medical Sciences, PAKISTAN

## Abstract

*Plasmodium* infection induces extensive remodeling of host erythrocytes through parasite-exported proteins that interact with host cell components*.* Among these, tryptophan-rich antigens (TRAgs) are conserved across *Plasmodium* species and have been proposed as vaccine candidates, but their molecular functions remain largely unexplored. In this study, we characterized *Plasmodium knowlesi* tryptophan-rich antigen 40.1 (PkTRAg40.1) to investigate its localization, biochemical properties, host interactions, and immunogenicity. Recombinant PkTRAg40.1 showed no detectable binding to intact human erythrocytes in flow cytometry-based binding assays. Biochemical characterization demonstrated that native PkTRAg40.1 was predominantly associated with detergent-resistant insoluble fractions and was detected as multiple higher-molecular-weight forms. Protease protection assays further showed that native PkTRAg40.1 was accessible to protease under the experimental conditions tested. Consistent with these observations, immunofluorescence assays revealed that PkTRAg40.1 colocalized with skeleton-binding protein 1 (PkSBP1), supporting its localization to Sinton and Mulligan’s clefts (SMCs), parasite-derived membranous structures. Pull-down assays and MALDI-TOF/MS identified spectrin alpha as a host binding partner, and biolayer interferometry confirmed its specific interaction with the spectrin alpha α12–16 fragment (K_D_ = 4.71 ± 0.62 μM). Serological analysis of *P. knowlesi* patient samples revealed that PkTRAg40.1 is immunogenic, exhibiting 50% sensitivity and 92.9% specificity, and also displayed cross-reactivity with sera from *Plasmodium vivax*-infected patients. Collectively, these findings identify PkTRAg40.1 as a TRAg associated with parasite-derived membranous structures that interacts with the host erythrocyte cytoskeleton, thereby expanding current understanding of the molecular functions of *Plasmodium* TRAgs.

## Author summary

Malaria parasites extensively remodel infected red blood cells by exporting proteins that interact with host cell components. Tryptophan-rich antigens (TRAgs) are conserved across *Plasmodium* species and are considered important for parasite biology, but their molecular functions remain unclear. Although previous studies have suggested that some TRAgs may associate with parasite-induced membrane structures and host cell structural components, the biological significance of these observations has yet to be established. We characterized PkTRAg40.1 from the zoonotic malaria parasite *P. knowlesi* to investigate its localization, host interactions, and immunogenicity. We found that recombinant PkTRAg40.1 showed no measurable binding to intact human erythrocytes in flow cytometry-based binding assays. Instead, PkTRAg40.1 localized to parasite-induced membranous structures within infected erythrocytes. Biochemical analyses identified spectrin alpha, a major component of the erythrocyte membrane skeleton, as a host binding partner. Patient sera further showed that PkTRAg40.1 is immunogenic and is recognized by antibodies from *P. knowlesi*-infected patients. To our knowledge, this is the first study to identify an interaction between a *P. knowlesi* TRAg and a host erythrocyte structural protein. These findings provide new molecular insight into the interaction of PkTRAg40.1 with host membrane and cytoskeletal components and provide a framework for future studies investigating the biological functions of TRAg proteins.

## Introduction

Malaria is a mosquito-borne infectious disease caused by protozoan parasites of the genus *Plasmodium*. According to the World Malaria Report 2025, there were an estimated 282 million cases and 610,000 deaths worldwide in 2024 [1]. Five *Plasmodium* species are known to cause malaria in humans, with *P. falciparum* and *P. vivax* accounting for the majority of cases [2]. Recently, the first malaria vaccine targeting *P. falciparum* was approved for malaria prevention and control. However, the vaccine is species-specific, and the high antigenic variability of the targeted antigens limits its efficacy by eliciting inconsistent antibody responses [3,4]. Given these limitations, there is an urgent need to identify novel antigens that are essential for parasite survival and conserved across species, to facilitate the development of more reliable vaccines.

The simian malaria parasite *P. knowlesi* has emerged as a significant zoonotic threat, with a growing number of naturally acquired human infections reported across Southeast Asia, particularly in Malaysia [5,6]. Phylogenetically, *P. knowlesi* and *P. vivax* are closely related, sharing a high degree of genetic similarity [7,8]. In both species, merozoite invasion involves key host-parasite interactions, including the binding of the Duffy-binding protein (DBP) to the Duffy antigen receptor for chemokines (DARC) and the involvement of reticulocyte-binding-like (RBL) proteins in erythrocyte invasion [9–11]. Additionally, tryptophan-rich antigens (TRAgs) identified in both *P. vivax* and *P. knowlesi* have been shown to interact with host erythrocytes [12]. Unlike *P. vivax*, which is challenging to culture, *P. knowlesi* can be continuously maintained *in vitro* using human erythrocytes. This makes it a valuable model for studying various biological aspects of *P. vivax* [13].

TRAgs are characterized by a conserved tryptophan-rich (TR) domain across *Plasmodium* species and have been proposed as potential vaccine candidates due to their strong immunogenicity and erythrocyte binding properties [4,14]. These antigens were first identified in *P. yoelii*, where they elicited strong immune responses in immunized hosts and were localized on the surface of infected erythrocytes [15,16]. In *P. falciparum*, TRAgs are referred to as tryptophan-threonine-rich antigens (PfTryThrA), comprising three family members. These proteins bind to human erythrocytes, and some have been shown to inhibit merozoite invasion through antibody-mediated mechanisms [17]. These proteins are expressed during the blood stage and localize at Maurer’s clefts, where they are closely associated with skeleton-binding protein 1 (PfSBP1), a key component of the parasite trafficking complex [18]. Interestingly, a total of 36 TRAg genes have been identified in *P. vivax*, reflecting a broader expansion within the *P. vivax* clade. These genes are organized in clusters along the chromosomes, suggesting gene duplication and diversification within this lineage [19,20]. Consistent with this genomic expansion, functional studies have demonstrated that ten PvTRAgs can bind to human erythrocytes, with some of them sharing the same receptor [4,21]. These TRAgs commonly interact with both host cell receptors and parasite proteins, reflecting their diverse functions for protein-protein interaction. For instance, PvTRAg38 binds to Band 3 and Basigin and facilitates merozoite invasion [22,23]. PvTRAg36.6 forms a complex with ETRAMP, suggesting a role in maintaining the parasitophorous vacuole membrane, while PvTRAg56.2 interacts with PvMSP7 to stabilize the merozoite surface complex during intraerythrocytic development [24]. Similar to *P. vivax*, *P. knowlesi* encodes a considerable number of TRAgs, totaling 26 genes. Of these, 20 proteins share more than 60% sequence identity, indicating a high degree of conservation between the two species [8,12,19]. This conservation suggests that TRAgs perform diverse biological roles in parasite biology, making them attractive targets for further investigation. From an immunological perspective, TRAgs contain conserved epitopes and exhibit high immunogenicity, eliciting both cellular and humoral immune responses [25,26]. In addition to their immunogenic potential, their subcellular localization provides further clues to their function. Localization studies suggest that in *P. vivax*-infected erythrocytes, TRAgs are localized near the caveola-vesicle complex (CVC), a unique structure observed in infected erythrocytes, implying a role in transporting parasite molecules from the erythrocyte cytoplasm to the extracellular environment [25,27,28].

Among the members of the *P. knowlesi* TRAg family, PkTRAg40.1 was selected for further investigation based on transcriptomic evidence supporting blood-stage expression and its high sequence conservation with the *P. vivax* ortholog, PVP01_0532700. Comparative transcriptomic and phylogenetic analyses suggest that PkTRAg40.1 belongs to an evolutionarily conserved TRAg lineage within the vivax clade, supporting the possibility that it retains conserved biological properties [29]. Previous studies have demonstrated that several TRAg family members participate in host-parasite interactions, including erythrocyte receptor recognition and protein-protein interactions [4,21–23]. More recently, certain TRAgs have also been shown to interact with specific membrane lipids and to exhibit structural features resembling BAR domain-containing proteins involved in membrane remodeling [19,30]. These findings suggest that the functional repertoire of TRAg proteins extends beyond direct erythrocyte receptor recognition and highlight the need for a detailed functional characterization of individual TRAg family members.

In this study, we investigated the subcellular localization, biochemical properties, host interaction partners, and antigenicity of PkTRAg40.1 during blood-stage *P. knowlesi* infection. We show that PkTRAg40.1 is associated with SMCs, exhibits biochemical characteristics consistent with a membrane-associated protein, interacts with erythrocyte spectrin alpha, and displays a detectable phosphatidic acid reactivity in a lipid dot blot assay. These findings expand our understanding of the functional diversity of TRAg proteins and provide new insights into the potential involvement of PkTRAg40.1 in membrane-associated host–parasite interactions.

## Materials and methods

### Ethics statement

All procedures adhered to relevant ethical standards and were approved by the Institutional Review Boards of Kangwon National University Hospital (IRB No. 2014-08-008-002), the University Malaya (Ref. No. 817.18), and the Malaysian Medical Research and Ethics Committee (MREC; NMRR-16-2840-33769). Written informed consent was obtained from all participants.

### Human sample collection and ethical compliance

Peripheral blood samples were obtained from *P. knowlesi*–infected patients in Malaysia, as previously described [31]. Control sera were collected from healthy children under 10 years of age residing in non-endemic regions of the Republic of Korea through Kangwon National University Hospital.

### Primary and tertiary structure analysis of PkTRAg40.1

The amino acid sequence of PkTRAg40.1 (PKA1H_050021800) from the *P. knowlesi* A1-H.1 strain was retrieved from PlasmoDB. Primary structural features, including domain organization and putative transmembrane regions, were analyzed using the Simple Modular Architecture Research Tool (SMART). For tertiary structure prediction, the full-length amino acid sequence was submitted to SWISS-MODEL and AlphaFold2. The AlphaFold2-predicted model was selected for further refinement using Galaxy Refine. Structural similarity was assessed by comparison with the *P. vivax* TRAg proteins PVP01_0000100 (PDB ID: 8ARL) and PVP01_0532700 using the DALI server, and structural alignments were visualized using UCSF ChimeraX version 1.8.

### Protein expression and purification

The PkTRAg40.1 protein construct was designed based on PlasmoDB (PKA1H_050021800.1). The gene encoding the tryptophan-rich (TR) domain (amino acids 67–325) was amplified from *P. knowlesi* A1-H.1 genomic DNA using gene-specific primers (S1 Table). The PCR product was cloned into the pProEX-HTB vector, which encodes an N-terminal 6 × His-tag, via *BamHI* and *XhoI* digestion and In-Fusion cloning (Clontech). Recombinant protein expression was induced in *E. coli* BL21(DE3), cultured in Luria-Bertani broth at an OD600 of 0.6–0.8, then treated with 0.1 mM IPTG at 37°C for 4 h. Cells were harvested (14,000 rpm, 20 min) and resuspended in PBS containing 50 mM HEPES, 163 mM NaCl, 5% glycerol, 1 mg/mL lysozyme, and 5 mM imidazole, adjusted to pH 6.8. The recombinant protein was purified using Ni Sepharose Excel resin (Cytiva), and purity was assessed by 12% SDS-PAGE followed by Coomassie brilliant blue staining.

Maltose-binding protein (MBP) was expressed in *E. coli* BL21(DE3) under the same conditions as PkTRAg40.1 (S2A Fig). To obtain soluble proteins, PkDBPα-RII and DARC were expressed in HEK293E cells using a pTT5-based vector (S2A and S2C Fig). His-tagged recombinant proteins were purified as previously described [32]. Human Fc-tagged proteins (IgG1-Fc fusion) were purified using a HiTrap Protein G HP column (Cytiva). Briefly, the culture supernatant from HEK293E cells was passed through the column twice, followed by two washes with 20 mM phosphate buffer (pH 7.0). Bound proteins were eluted in five fractions using 1 mL of 0.1 M glycine-HCl (pH 2.7), and each eluate was immediately neutralized with 70 μL of 1 M Tris-HCl (pH 9.0).

### Generation of polyclonal antibodies

Female BALB/c mice were immunized via the intraperitoneal (i.p.) route with 30 µg of purified PkTRAg40.1 emulsified in Freund’s complete adjuvant (Sigma). Freund’s incomplete adjuvant was used for three subsequent booster injections administered at three-week intervals. Serum was collected via cardiac puncture two weeks after the final immunization.

### Parasite culture

*P. knowlesi* A1-H.1 parasites were maintained in human erythrocytes in RPMI-1640 medium at 37°C under a gas mixture of 90% N_2_, 5% O_2_, and 5% CO_2_. The 28-h intraerythrocytic cycle was maintained by replacing the culture medium every 14 h. Parasitemia was monitored using Giemsa-stained blood smears. Parasite synchronization and enrichment of late trophozoite and schizonts were performed using magnetic-activated cell sorting (MACs) LD columns (Miltenyi Biotec).

### Protein gel electrophoresis and immunoblotting

SDS-PAGE and immunoblotting were performed to evaluate the quality of purified recombinant proteins and native proteins in parasite lysates. Recombinant proteins used in this study consisted of His-tagged PkTRAg40.1 and GST-tagged spectrin alpha fragments. Purified recombinant proteins were separated on 12% Tris-Glycine gels, whereas native parasite lysate samples were resolved on 4–12% Bolt Bis-Tris gels (Thermo Fisher), following denaturation in reducing sample buffer. Proteins were transferred to 0.45 μm nitrocellulose membranes using the Mini Gel Tank and Blot Module Set (Thermo Fisher). Membranes were blocked overnight at 4°C in PBS-T containing 5% skim milk and subsequently incubated with the following primary antibodies: mouse anti-His monoclonal antibody (1:2,000; Abclonal), mouse anti-GST monoclonal antibody (1:5,000), mouse anti-PkTRAg40.1 serum (1:50), rabbit anti-PkSBP1 serum (1:50), rabbit anti-spectrin alpha antibody (1:1,000; Abclonal), and mouse anti-*Plasmodium* aldolase antibody (1:1,000; Abnova). After washing, membranes were incubated with IRDye 800CW goat anti-mouse IgG or IRDye 800CW goat anti-rabbit IgG secondary antibodies (LI-COR Biosciences, Lincoln, NE, USA) and analyzed using an Odyssey infrared imaging system (LI-COR Biosciences).

For Blue Native PAGE (BN-PAGE), purified recombinant PkTRAg40.1 was mixed with NativePAGE sample buffer and G-250 sample additive (Thermo Fisher) and separated on a 4–16% NativePAGE gel (Thermo Fisher) under non-denaturing conditions according to the manufacturer's instructions. Protein bands were visualized by Coomassie Brilliant Blue staining.

### Protein solubility analysis

Protein solubility analysis was performed as previously described [33]. Briefly, purified late trophozoite-stage infected erythrocytes were subjected to hypotonic lysis in ice-cold 2 mM Tris-HCl containing protease inhibitors. Following centrifugation, the membrane pellet was sequentially extracted with 0.1 M sodium carbonate (pH 11), 5 M urea, or 1% Triton X-100 in PBS. Soluble and insoluble fractions were separated by centrifugation, and all fractions were analyzed by SDS-PAGE followed by Western blotting.

### Protease protection assay

Protease protection assays were performed based on previously described methods with minor modifications [33]. Briefly, magnet-purified late trophozoite-stage infected erythrocytes were resuspended in PBS and treated with either 0.05% saponin or 3 U streptolysin O (SLO) for 10 min at room temperature with shaking. Samples were subsequently incubated with proteinase K (20 μg mL^-1^ final concentration) for 20 min at 37°C. Proteolysis was terminated by the addition of 1 mM PMSF and Complete Protease Inhibitor Cocktail (Roche), followed by incubation for 3 min and washing with ice-cold PBS. After centrifugation (5,000 × g, 5 min, 4°C), the pellets were resuspended in reducing sample buffer and analyzed by SDS-PAGE and Western blotting.

### Lipid dot blot assay

Lipid-binding activity was assessed using PIP Strips (Thermo Fisher) according to the manufacturer's instructions with minor modifications. Membranes were blocked overnight at 4°C with 3% fatty acid-free bovine serum albumin (Sigma, A4612) in TBS-T. The membranes were then incubated with 1 μg of purified recombinant His-tagged PkTRAg40.1 or His-tagged maltose-binding protein (MBP), used as a negative control, for 2 h at RT. After washing three times with TBS-T, membranes were detected using a mouse anti-His monoclonal antibody (1:2,000; Abclonal) followed by IRDye 800CW goat anti-mouse IgG (1:10,000; LI-COR Biosciences) diluted in TBS-T. Fluorescence signals were visualized using an Odyssey infrared imaging system (LI-COR Biosciences).

### Indirect immunofluorescence assay (IFA)

Mixed-stage parasites were fixed on slides with 4% paraformaldehyde and blocked with 5% BSA. Slides were incubated with mouse anti-PkTRAg40.1 (1:100), rabbit anti-PkSBP1 (1:100), or rabbit anti-spectrin alpha (1:200, Abclonal) antibodies at 37°C for 1 h. Secondary antibodies used were Alexa Fluor 488-conjugated goat anti-mouse IgG and Alexa Fluor 594-conjugated goat anti-rabbit IgG (1:500, Invitrogen). Nuclei were stained with DAPI (1:1,000) at 37°C for 30 min. Slides were mounted using ProLong Gold Antifade Mountant (Invitrogen) and visualized using a FLUOVIEW FV3000 confocal microscope (Olympus). Image analysis and Pearson's correlation coefficient calculations were performed using FV31S-SW_2.6 and SigmaPlot v12, respectively.

### Protein microarray for humoral immune response

Three aminopropyl-coated slides were spotted with recombinant PkTRAg40.1 protein (50 ng/µL) and incubated at 37°C for 2 h. After blocking with 5% bovine serum albumin (BSA) for 1.5 h, the slides were incubated in duplicate with serum samples from *P. knowlesi*-infected patients (Malaysia, n = 70), *P. vivax*-infected patients (Republic of Korea, n = 70), and healthy individuals (n = 42), diluted 1:25 in PBS-T at 37°C for 1.5 h. The antigenic reactivity was detected using Alexa Fluor 546-conjugated goat anti-human IgG (10 ng/µL, Invitrogen). The slides were scanned using an InnoScan 300-G Microarray scanner (Innopsys), and fluorescence intensity was quantified using MAPIX v7.4.1 software. The seropositivity cut-off value was defined as the mean fluorescence intensity of negative controls plus two standard deviations. Protein array analyses of recombinant PkMSP1–19 and PkSBP1 proteins were performed using the same experimental procedure with the *P. knowlesi*-infected serum panel to evaluate the comparative seroreactivity. Receiver operating characteristic (ROC) curve analysis was performed using GraphPad Prism version 8.0.2 to further evaluate the antibody response profiles.

### Erythrocyte binding assay by flow cytometry

Erythrocytes (1 × 10^6^ cells/mL) were incubated with 0–40 μg/mL of recombinant PkTRAg40.1 protein at 25 °C for 3 h. PkDBPα-RII and MBP served as positive and negative controls, respectively. After incubation, cells were washed three times with 200 μL of PBS containing 1% BSA, then stained with mouse anti-penta-His monoclonal antibody conjugated to Alexa Fluor 647 (Qiagen) at 25 °C for 1 h in the dark condition. The samples were washed three times with 1% BSA-PBS, and fluorescence was measured using a CytoFLEX flow cytometer (Beckman Coulter), acquiring 200,000 events per sample. Data was analyzed using CyExpert software, v2.6 (Beckman Coulter).

### Co-affinity purification and mass spectrometry

His-tagged PkTRAg40.1 was immobilized on HisPur Cobalt resin (Thermo Fisher) at 4°C for 3 h. Erythrocyte membranes were prepared by hypotonic lysis of washed erythrocytes in ice-cold 10 mM Tris-HCl (pH 7.4), followed by repeated centrifugation and washing until white ghost membrane pellets were obtained. The ghost membrane pellets were lysed in Pull-Down Lysis Buffer (Thermo Fisher) supplemented with a 1 × protease inhibitor cocktail on ice for 30 min and centrifuged at 20,000 × g for 30 min at 4°C. The resulting supernatant containing erythrocyte membrane proteins was collected, and 900 µg of lysate was incubated with the immobilized bait protein overnight at 4°C [34]. After extensive washing, bound proteins were eluted and mixed with 4X Bolt LDS Sample Buffer (Thermo Fisher) and 10X Bolt Sample Reducing Agent (Thermo Fisher) at a final 1 × concentraion, heated at 95°C for 5 min, separated via Bolt 4–12% Bis-Tris gels (Thermo Fisher), and visualized by silver staining (Pierce Silver Stain Kit). Protein bands were excised, subjected to trypsin digestion, and analyzed using MALDI-TOF MS (Genomine Co., Ltd). Spectra (700–4000 m/z) were internally calibrated using trypsin peaks. Protein identification was performed using the MASCOT search engine, and resulting spectra were analyzed with FlexAnalysis v3.4.

### Recombinant spectrin alpha fragment preparation

To overcome challenges in expressing full-length spectrin alpha (~280 kDa), the protein was divided into four overlapping fragments (αN-5, α6–11, α12–16, α17–C) amplified from a cDNA ORF clone using specific primers (S1 Table) [35]. PCR products were cloned into the pGEX4T-1 vector and transformed into *E. coli* BL21(DE3). Expression was induced with 0.5 mM IPTG at 37°C for 4 h. Soluble proteins were purified using Glutathione Sepharose 4B (Cytiva), followed by buffer exchange into PBS using 10 kDa Amicon spin columns (Millipore).

### Pull-down assays

His-tagged PkTRAg40.1 (50 µg) was immobilized on Ni-NTA resin (Qiagen) and incubated with individual spectrin alpha fragments. Following extensive washing, protein complexes were eluted and analyzed by Coomassie staining and Western blotting. To validate interactions under native proteins, synchronized late trophozoite- and schizont-stage parasites were lysed in 1% Triton X-100-containing lysis buffer as described in the protein solubility assay. Following centrifugation, the Triton X-100-soluble fraction was collected and incubated with GST-tagged spectrin alpha fragments α6–11 and α12–16 immobilized on Glutathione Sepharose 4B resin (Cytiva). After extensive washing, bound proteins were eluted and analyzed by immunoblotting.

### Binding kinetics by Biolayer interferometry (BLI)

The binding kinetics between PkTRAg40.1 and spectrin alpha fragments (α6–11, α12–16) were assessed using a biolayer interferometry system (Gator Bio) with Ni-NTA biosensors. His-tagged PkTRAg40.1 was immobilized on the biosensor surface and then incubated with two-fold serial dilutions of spectrin alpha fragments in kinetic buffer (0.02% Tween-20 in PBS, pH 7.4). Data were analyzed using GatorOne v2.15 software with a 1:1 binding model. A known interaction pair, PkDBPα-RII and DARC, served as the positive control and was modeled using a 2:1 binding curve. All BLI experiments were performed in three independent experiments. Equilibrium dissociation constants (KD) are presented as mean ± standard deviation (SD). Kinetic parameters were obtained by global fitting of the sensorgrams, and the quality of the fitting was evaluated using the coefficient of determination (R^2^).

### Statistical analysis

All statistical analyses were performed using GraphPad Prism version 8.0.2. (GraphPad Software, San Diego, USA). Data are presented as mean ± standard deviation (SD) unless otherwise indicated. Group comparisons were performed using unpaired two-tailed Student's *t*-tests. A *p*-value < 0.05 was considered statistically significant (*p < 0.05, **p < 0.01, ***p < 0.001). For serological analyses, the cutoff value was defined as the mean fluorescence intensity (MFI) of the healthy control group plus two standard deviations (mean + 2 SD). Diagnostic sensitivity and specificity were calculated based on this cutoff value, and 95% confidence intervals (95% CIs) were determined using the exact binomial method. Biolayer interferometry (BLI) kinetic parameters were calculated using the GatorOne software by global fitting of the binding curves to the indicated interaction model, and the goodness-of-fit was evaluated using the coefficient of determination (R^2^).

## Results

### Structural characterization and expression of PkTRAg40.1

**Fig 1:**
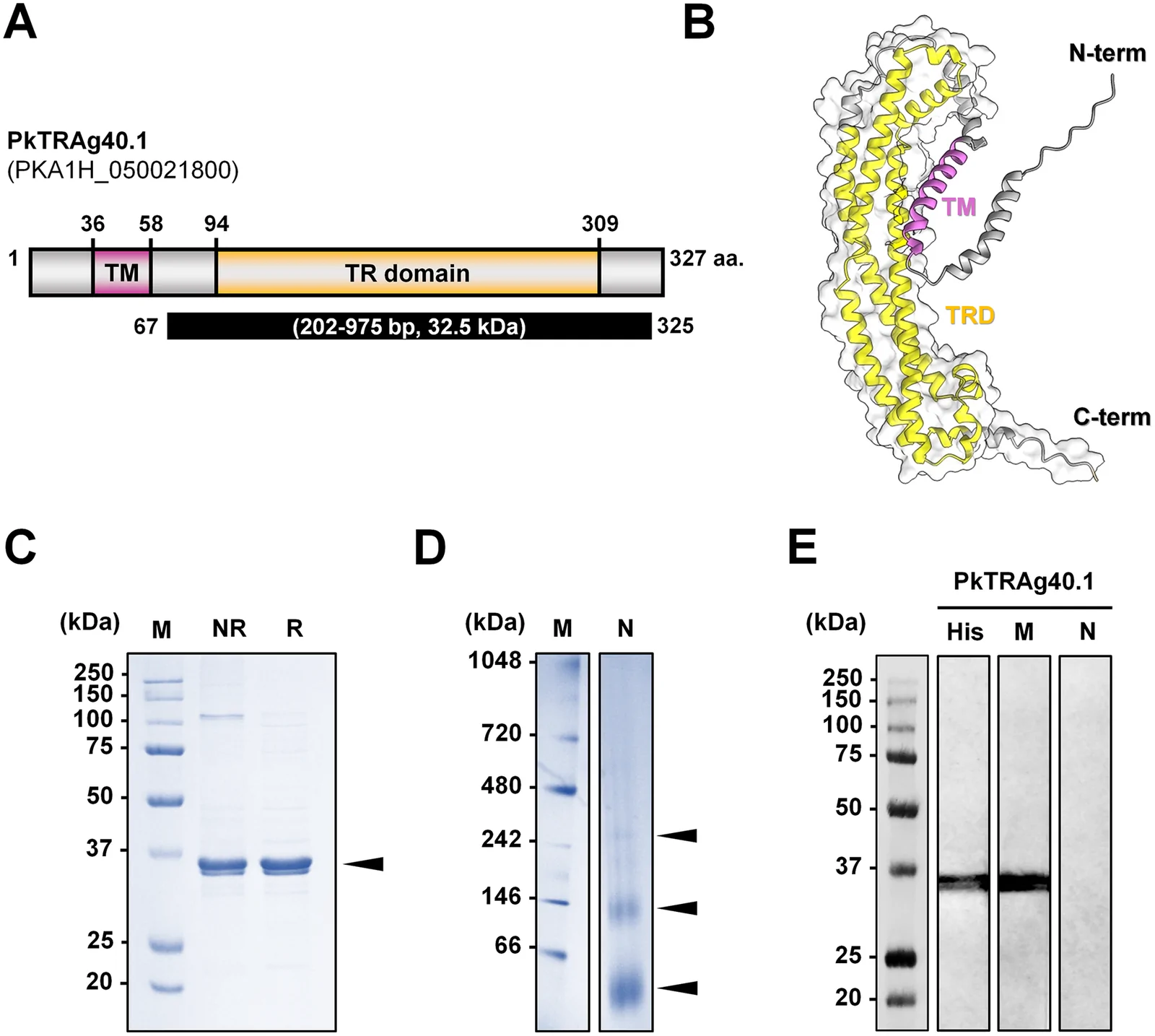
Schematic structure, recombinant expression, and specific antibody production of PkTRAg40.1. **(A)** Schematic representation of PkTRAg40.1. The sequences used for cloning and expression of the recombinant protein PkTRAg40.1 (amino acids 67–325) are indicated by black lines. The transmembrane domain (purple box) and tryptophan-rich (TR) domain (yellow box) are highlighted. **(B)** AlphaFold2-predicted tertiary structure of PkTRAg40.1. The transmembrane domain (TM) and TR domain are highlighted in purple and yellow, respectively. The recombinant protein region (67-325 aa) is shown as a surface representation. **(C)** Coomassie Brilliant Blue-stained SDS-PAGE analysis of purified recombinant PkTRAg40.1 under non-reducing (NR) and reducing (R) conditions. **(D)** Blue Native PAGE (BN-PAGE) analysis of recombinant PkTRAg40.1 under non-denaturing conditions, revealing multiple molecular weight bands. NativeMark Unstained Protein Standard (M), recombinant PkTRAg40.1 (N). **(E)** Western blot analysis of the purified recombinant PkTRAg40.1 protein under reducing conditions using mouse anti-His tag monoclonal antibody (His), immune sera (M), and pre-immune sera (N).

The *pktrag40.1* gene (PKA1H_050021800) is located on chromosome 5, spans 1,262 base pairs (bp), and contains a single intron. It encodes a 326-amino acid (aa) protein with a predicted molecular weight of 40.1 kDa. Primary structural analysis revealed the presence of a transmembrane domain (36–58 aa) and a tryptophan-rich (TR) domain (94–309 aa) (Fig 1A). AlphaFold2-based structural prediction revealed that PkTRAg40.1 adopts an elongated α-helical architecture. Electrostatic surface analysis further identified a prominent positively charged patch at the N-terminal region of the recombinant TR domain (S1B Fig). The recombinant protein region used in this study (residues 67–325), encompassing the predicted tryptophan-rich (TR) domain, is highlighted as a white surface representation in the model (Figs 1B and S1A). Based on the predicted domain organization, this region was selected for recombinant protein expression and subsequent functional analyses. The TR domain is a conserved erythrocyte-binding motif among TRAgs across *Plasmodium* species. Structural comparison of the TRD region revealed a high degree of conservation between PkTRAg40.1 and previously characterized PvTRAg proteins. DALI analysis further confirmed that PkTRAg40.1 shares a conserved structural fold with PVP01_0000100 and PVP01_0532700, with RMSD values of 1.6 Å and 0.8 Å and Z-scores of 20.4 and 23.8, respectively (S1C Fig). A recombinant protein comprising the TR domain (67–325 aa) was successfully expressed in *E. coli* and purified as a soluble form, exhibiting the expected molecular weight of 35.9 kDa, including the tag (Fig 1C). Blue Native PAGE (BN-PAGE) was performed under non-denaturing conditions. Recombinant PkTRAg40.1 migrated as multiple molecular weight forms rather than a single band, with prominent signals detected at approximately 20–66 kDa, ~ 146 kDa, and ~242 kDa (Fig 1D). The detection of these higher-molecular-weight bands indicates that recombinant PkTRAg40.1 adopts heterogeneous molecular forms under native conditions rather than existing exclusively as a monomer. The purity of the recombinant protein was confirmed by western blotting using an anti-His-tag antibody (Fig 1E, Lane His). Immunization with the purified protein generated polyclonal antibodies, which specifically recognized recombinant PkTRAg40.1 (Fig 1E, Lane M), while pre-immune sera showed no reactivity (Fig 1E, Lane N).

### Biochemical properties of native PkTRAg40.1

**Fig 2:**
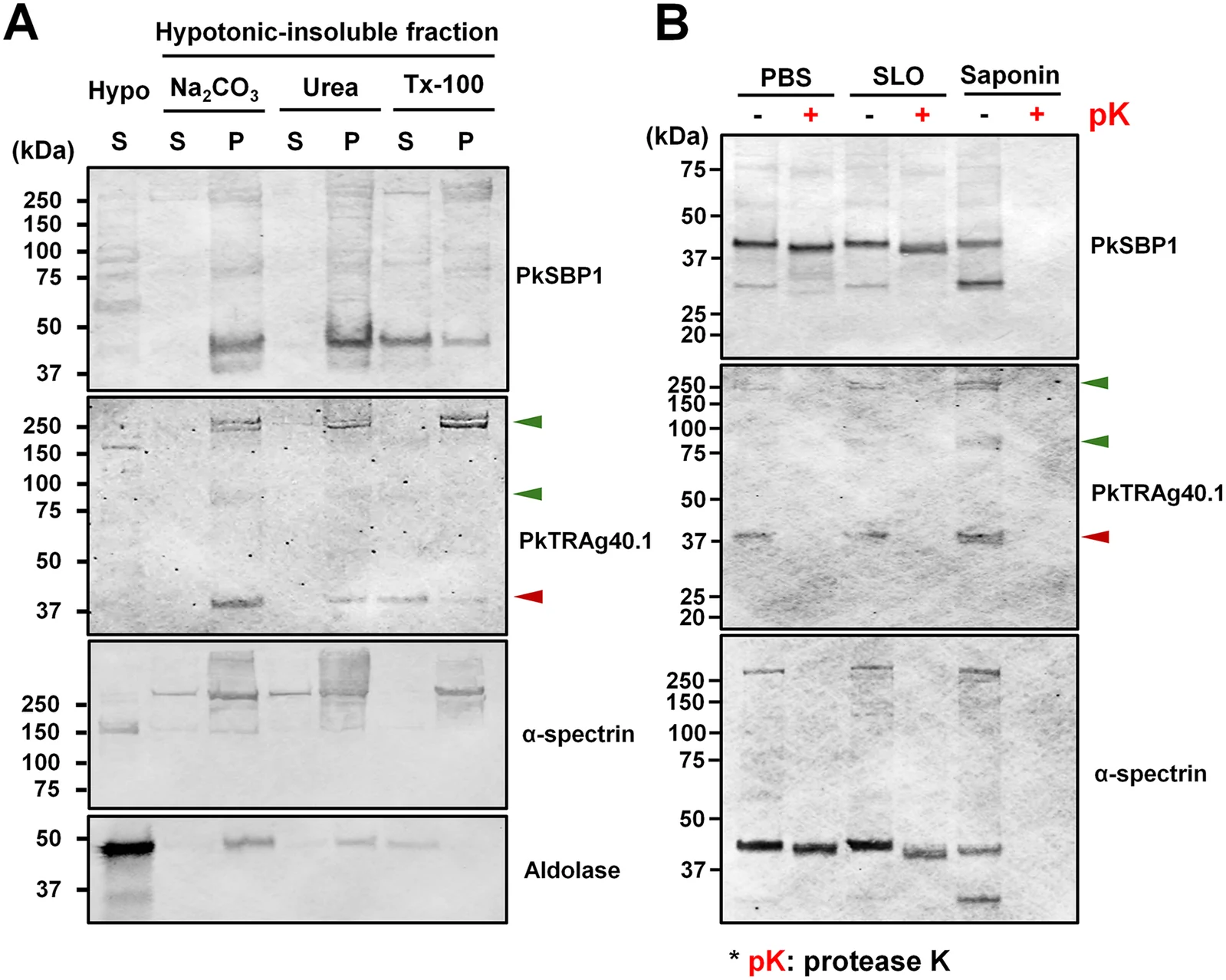
Solubility analysis and protease accessibility of PkTRAg40.1. (**A)** Protein solubility analysis of PkTRAg40.1 in *P. knowlesi* A1-H.1-infected erythrocytes. After hypotonic release of soluble proteins (Hypo), the hypotonic insoluble fractions were sequentially treated with sodium carbonate (Na_2_CO_3_), 5M urea, and 1% Triton X-100. Supernatant (S) and pellet (P) fractions from each extraction step were collected. **(B)** Protease protection assay of PkTRAg40.1. Infected erythrocytes were treated with PBS, streptolysin O (SLO), or saponin in the presence (+) or absence (−) of proteinase K (pK). Insoluble fractions were subsequently prepared. PBS preserves both the erythrocyte and parasitophorous vacuole membranes (PVM), SLO selectively permeabilizes the erythrocyte membrane, and saponin disrupts the PVM while largely preserving the parasite plasma membrane. For both assays, samples were analyzed by immunoblotting using anti-PkSBP1 rabbit sera, anti-PkTRAg40.1 mouse sera, anti-spectrin alpha polyclonal antibody, and anti-aldolase polyclonal antibody. Red arrowheads indicate the expected ~ 40 kDa PkTRAg40.1 band, whereas green arrowheads indicate additional higher-molecular-weight immunoreactive bands consistently detected across the assays. Uncropped western blots are shown in S3 and S4 Figs.

To investigate the biochemical properties of native PkTRAg40.1, sequential protein solubility extractions were performed using *P. knowlesi* A1-H.1-infected parasite lysates. Unlike the soluble parasite protein aldolase, which was predominantly detected in the hypotonic soluble fraction, PkTRAg40.1 was not detected in the hypotonic soluble fraction but was mostly present in the insoluble pellet fractions. Within the insoluble fractions, PkTRAg40.1 remained largely resistant to extraction with sodium carbonate and urea and was only partially solubilized by Triton X-100 treatment, indicating that native PkTRAg40.1 exhibits a strong association with detergent-resistant insoluble components (Figs 2A and S3).

Immunoblot analysis detected native PkTRAg40.1 protein signals at approximately ~ 40 kDa, 75–100 kDa, and ~250 kDa. In contrast, PkSBP1, a cellular marker of parasite-derived membranous structures known as SMCs, exhibited greater solubility under the same extraction conditions, whereas the host cytoskeletal protein spectrin alpha showed a solubility profile similar to that of PkTRAg40.1. These results indicate that native PkTRAg40.1 is predominantly associated with insoluble membrane-associated fractions in *P. knowlesi*-infected erythrocytes.

To investigate the subcellular accessibility and compartmental organization of native PkTRAg40.1 in *P. knowlesi*-infected erythrocytes, a protease protection assay was performed using insoluble fractions prepared from PBS-, SLO-, and saponin-treated samples. The native PkTRAg40.1 bands were no longer detected following proteinase K treatment under all permeabilization conditions, indicating that PkTRAg40.1 was accessible to protease under the experimental conditions tested. However, these data do not distinguish whether PkTRAg40.1 resides within the parasitophorous vacuole, the erythrocyte cytosol, or parasite-induced membranous compartments (Figs 2B and S4). Notably, in the absence of protease K, native PkTRAg40.1 was preferentially detected in saponin treatment samples, whereas weaker signals were observed under PBS and SLO treatment, suggesting that detergent-mediated membrane solubilization improves recovery of native PkTRAg40.1.

To further characterize the membrane-associated properties of PkTRAg40.1, its lipid-binding activity was assessed using PIP strips containing a panel of representative phospholipids and phosphoinositide. Recombinant His-tagged PkTRAg40.1 exhibited detectable reactivity toward phosphatidic acid (PA) in the lipid dot blot assay, whereas no signal was observed with the His-tagged MBP negative control (S5 Fig). No appreciable binding was detected for the other lipids represented on the lipid strips.

### Localization of PkTRAg40.1 to Sinton and Mulligan’s clefts, serological profiling, and lack of erythrocyte-binding activity

**Fig 3:**
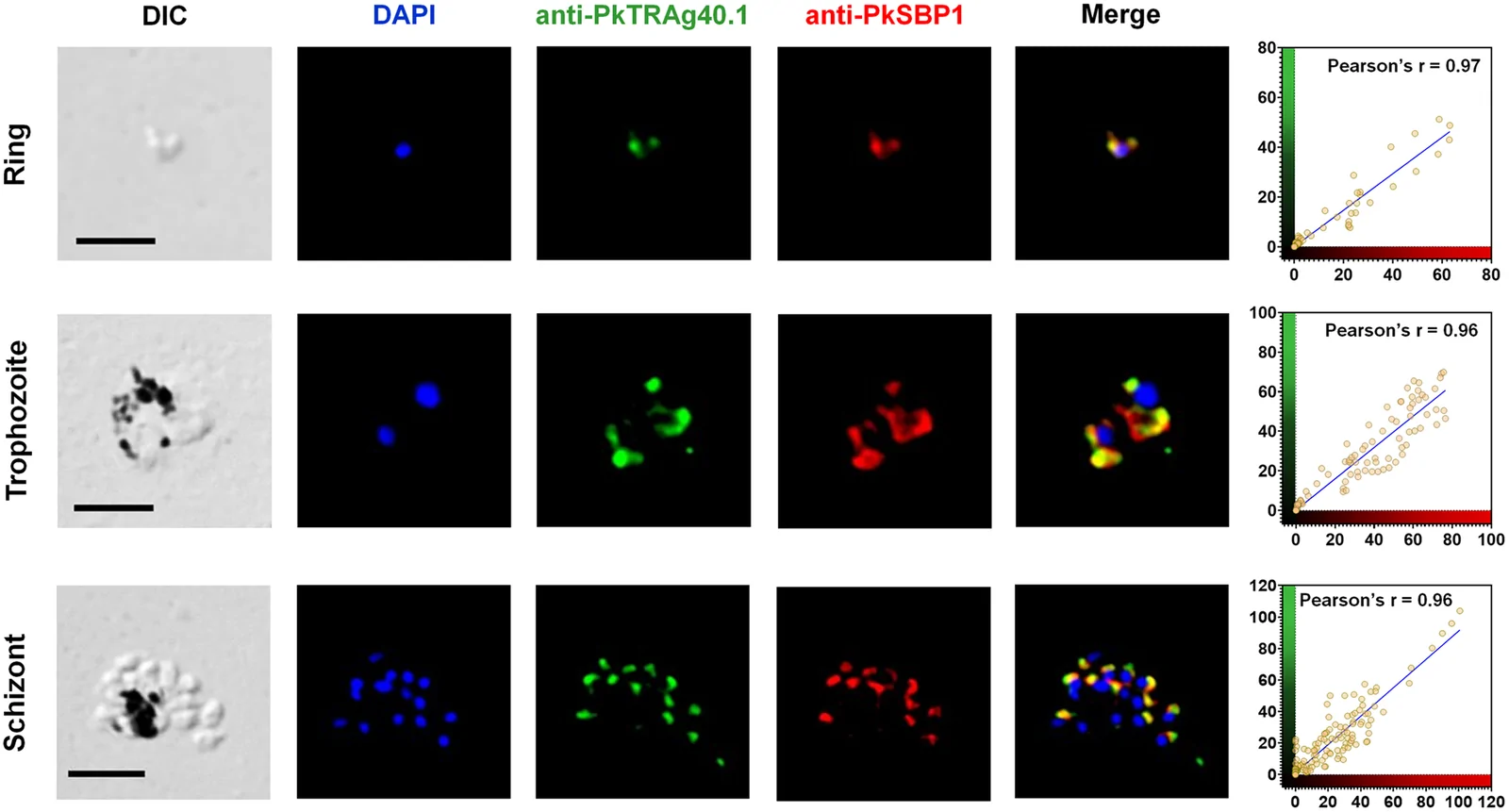
Subcellular localization of PkTRAg40.1 in asexual blood-stage parasites. Immunofluorescence assays were performed on *P. knowlesi* A1-H.1 parasites at various asexual blood stages. Parasites were probed with mouse immune serum against PkTRAg40.1 (green) and co-labeled with rabbit immune serum against PkSBP1 (red), a cleft-associated marker. Parasite nuclei were stained with DAPI (blue). DIC, differential interference contrast. Scale bar, 5 μm. Quantitative analysis of signal overlap was performed by correlation analysis of fluorescence intensities using SigmaPlot 12.0 and GraphPad Prism. Correlations were assessed using Pearson’s correlation coefficients (r) are shown in the scatter plots.

The subcellular localization of PkTRAg40.1 during the asexual blood stage of *P. knowlesi* was assessed via immunofluorescence assay. Co-staining with PkSBP1, a marker of Sinton and Mulligan’s clefts, demonstrated a high degree of colocalization, suggesting that PkTRAg40.1 is exported to these parasite-induced membranous structures (Fig 3). The pixel intensity correlation analysis yielded Pearson’s correlation coefficients of r = 0.97 and r = 0.96 for the ring and trophozoite stages, respectively (Fig 3). In contrast, weaker colocalization was observed with markers of the merozoite surface (PkMSP1–19, r = 0.69), micronemes (PkDBPα, r = 0.80), and rhoptry bulbs (PkRAMA, r = 0.69) (S6A–S6C Fig). PkTRAg40.1 displayed a punctate localization pattern during the ring stage, which became progressively redistributed into multiple dispersed foci during trophozoite and schizont development (S6D Fig).

**Fig 4:**
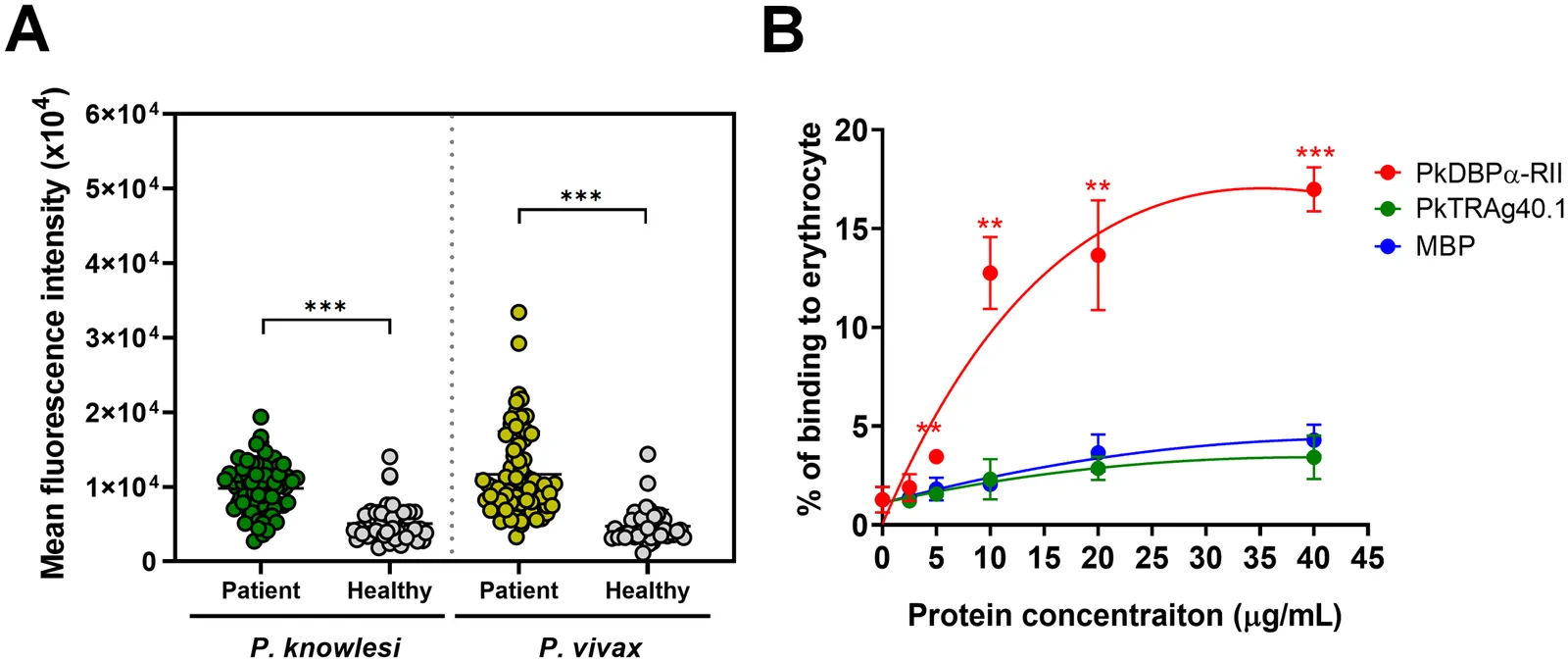
Antigenicity and host cell surface interaction activity of PkTRAg40.1. **(A)** Antigenicity of PkTRAg40.1. Total IgG responses to recombinant PkTRAg40.1 were measured by protein microarray using sera from *P. knowlesi*-infected patients (n = 70), *P. vivax*-infected patients (n = 70), and uninfected healthy individuals (n = 42). Bars indicate mean fluorescence intensity (MFI) ± standard deviation (SD). Statistical significance was assessed using Student’s *t*-test. **(B)** Erythrocyte-binding activity of His-tagged recombinant PkTRAg40.1 was evaluated by flow cytometry. Up to 1 × 10^6 human erythrocytes were incubated with increasing concentrations of recombinant protein (0–40 μg/mL). PkDBPα-RII and maltose-binding protein (MBP) were used as positive and negative controls, respectively. Data represent the mean ± SD of four independent experiments.

Serological profiling using a protein microarray revealed that PkTRAg40.1 is antigenic in naturally infected individuals. Among 70 *P. knowlesi*-infected patients and 42 healthy controls, PkTRAg40.1 exhibited a seroprevalence of 50.0% (95% CI: 38.6–61.4%) and a specificity of 92.9% (95% CI: 80.9–97.5%) (Table 1). Total IgG responses against PkTRAg40.1 were significantly elevated in *P. knowlesi*-infected patient sera compared with healthy controls (*p* < 0.0001) (Fig 4A). Although the antibody response to PkTRAg40.1 was lower than that observed for the merozoite surface antigen PkMSP1–19, it was comparable to that of PkSBP1 (Table 1 and S7A Fig). To evaluate potential cross-reactivity, sera from 70 *P. vivax*-infected patients were additionally analyzed. Total IgG responses against PkTRAg40.1 were significantly elevated in *P. vivax*-infected patients compared with healthy controls (*p* < 0.0001) (Fig 4A). Sequence alignment and structural analyses revealed a high degree of conservation between PkTRAg40.1 and the orthologous *P. vivax* TRAg (PVP01_0532700) (S1B and S1C Figs). In addition, predicted surface-exposed antigenic regions were conserved between the two proteins (S7C Fig). Receiver operating characteristic (ROC) analysis was performed to assess the discriminatory performance of PkTRAg40.1. The analysis yielded AUC values of 0.878 (95% CI: 0.806–0.948) and 0.930 (95% CI: 0.879–0.981) for *P. knowlesi* and *P. vivax* sera, respectively (S7B Fig). To assess the erythrocyte-binding properties of PkTRAg40.1, a flow cytometry–based binding assay was performed. PkDBPα-RII and MBP were used as positive and negative controls, respectively. PkDBPα-RII showed significant binding to human erythrocytes compared to MBP, whereas PkTRAg40.1 exhibited no detectable binding activity (Fig 4B). PkDBPα-RII binding became apparent at 5 μg/mL and reached saturation at 40 μg/mL, with a maximum binding level of 17.0 ± 1.1%.

**Table 1:** Serological reactivity to PkTRAg40.1, PkMSP1-19, and PkSBP1 in naturally acquired malaria infections.

Antigens (*P. knowlesi*)	Sample(n)	No. of Samples		Sensitivity (%)^a^/ Specificity (%)^b^	95% CI (%)^c^	MFI^d^	*p-*value^e^
**Positive**	**Negative**
**PkMSP1–19**	*P. knowlesi* patient (70)	50	20	71.4^a^	59.9-80.6	10148	*p* < 0.0001
	Healthy (42)	1	41	97.6^b^	87.6-99.5	3750	
**PkSBP1**	*P. knowlesi* patient (70)	29	41	41.4^a^	30.6-53.1	9783	*p* < 0.0001
	Healthy (42)	3	39	92.9^b^	80.9-97.5	3523	
**PkTRAg40.1**	*P. knowlesi* patient (70)	35	35	50.0^a^	38.6-61.4	9784	*p* < 0.0001
	Healthy (42)	3	39	92.9^b^	80.9-97.5	5080	
	*P. vivax* patient (70)	40	30	57.1^a^	45.5-68.1	11663	*p* < 0.0001
	Healthy (42)	2	40	95.2^b^	84.2-98.9	4711	

a Sensitivity/ seropositivity rate: percentage of positive results in malaria patient samples.

b Specificity/ seronegativity rate: percentage of negative in healthy samples.

c CI: Confidence intervals.

d MFI, mean fluorescence intensity was divided by the cutoff value +2 standard deviations above the MFI of healthy samples.

e *p*-values were determined using Student's *t*-test by comparing total IgG responses between *P. knowlesi*-infected patients and healthy controls. A *p*-value of <0.05 was considered statistically significant.

### Interaction between host cytoskeletal protein spectrin alpha (SPTA1) and PkTRAg40.1

**Fig 5:**
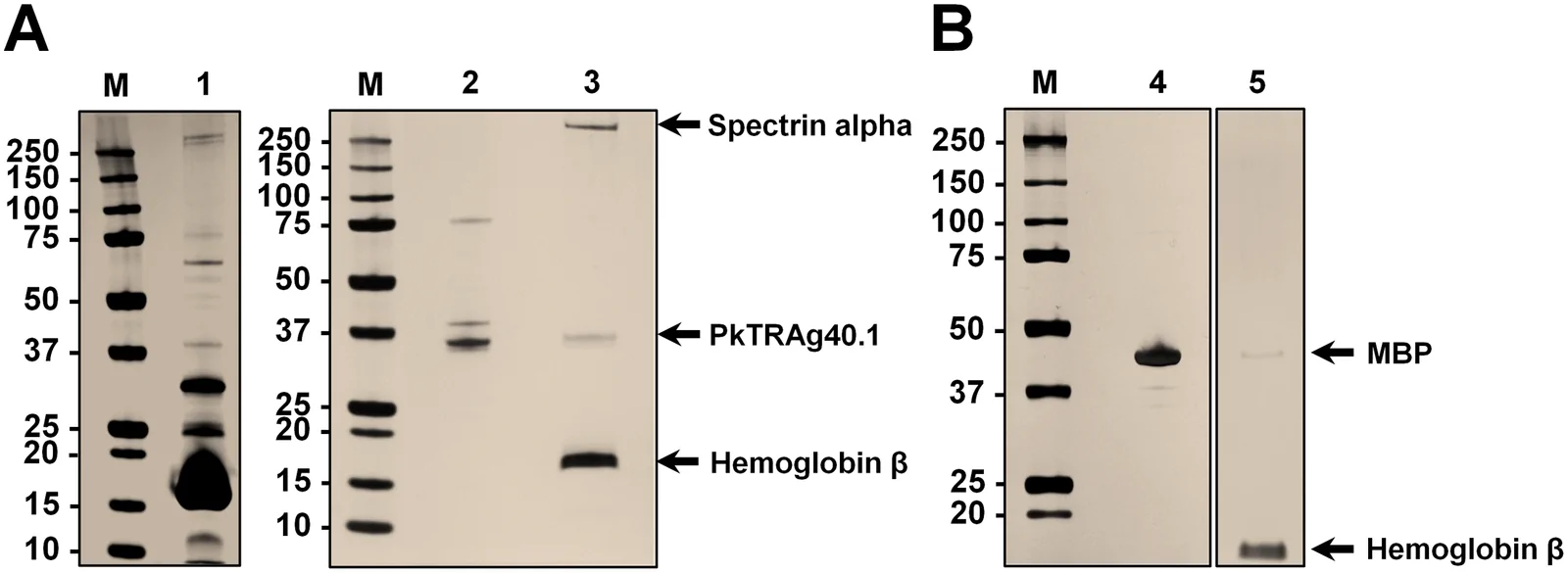
Erythrocyte proteins pulled down by recombinant PkTRAg40.1. Silver-stained Bis-Tris 4–12% gels showing proteins pulled down from human erythrocyte lysates by His-tagged PkTRAg40.1 **(A)** or maltose-binding protein (MBP; **B)**. Lane M, molecular weight marker; Lane 1, erythrocyte proteins; Lane 2, His-tagged PkTRAg40.1; Lane 3, erythrocyte proteins incubated with PkTRAg40.1; Lane 4, His-tagged MBP; Lane 5, erythrocyte proteins incubated with MBP. In panel A (Lane 3), bands corresponding to spectrin alpha, PkTRAg40.1, and hemoglobin β were detected. In panel B (Lane 5), bands corresponding to MBP and Hemoglobin β were observed.

To identify host proteins interacting with PkTRAg40.1, co-affinity purification was performed using His-tagged recombinant protein immobilized on resin, with erythrocyte membrane lysates as prey. Eluted fractions were resolved by Bis-Tris PAGE and visualized by silver staining, revealing specific bands (Fig 5A). MALDI-TOF/MS analysis of the excised bands identified peptides corresponding to spectrin alpha (SPTA1) and hemoglobin subunit beta (hemoglobin β) (Tables 2, S2). Given the detection of hemoglobin β in negative control MBP pull-downs, this interaction was considered non-specific (Fig 5B), whereas spectrin alpha was identified as a specific interactor. To further assess nonspecific interactions with erythrocyte membrane proteins, GST was used as an additional negative control in an independent pull-down assay (S8 Fig).

**Table 2:** Identification of erythrocyte membrane proteins binding to PkTRAg40.1 from the silver staining gel and obtained from the MALDI-TOF analysis and Mascot PMF search.

No	Name of protein	Accession no.	Data base	Molecular Mass [kDa]	Significance	Sequence coverage (%)	Mascot score
**1**	Spectrin alpha chain, erythrocytic 1	P02549	Swiss -Prot	279.842	*p* < 0.05	12	104
**2**	Hemoglobin subunit β	P68871	Swiss -Prot	15.988	*p* < 0.05	74	114

### Characterization of the interaction between spectrin alpha and PkTRAg40.1

**Fig 6:**
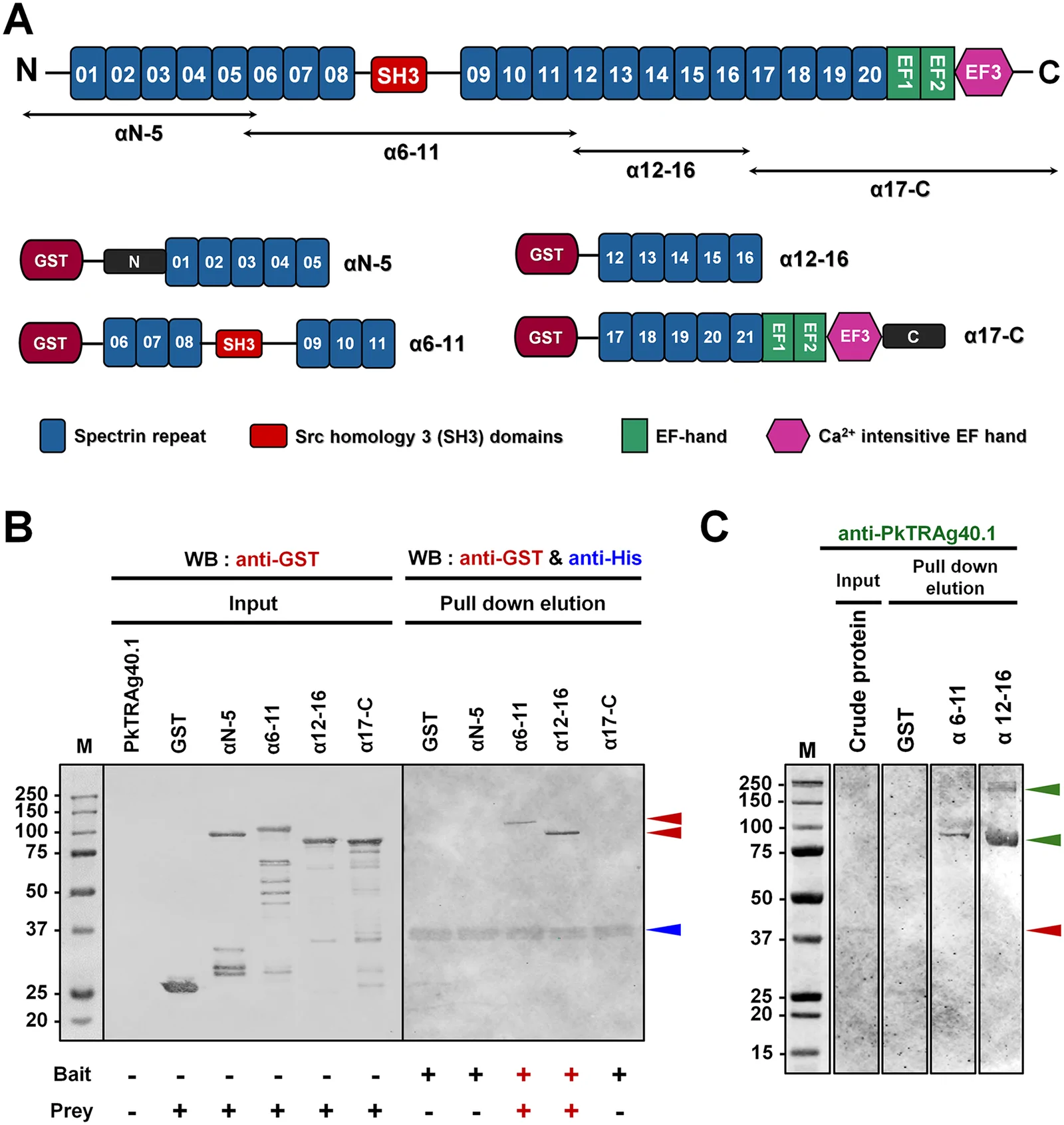
Identification of PkTRAg40.1 binding sites within human erythrocyte spectrin alpha. **(A)** Schematic representation of spectrin alpha showing spectrin repeats (blue), SH3 domain (red), EF-hand motifs (green), and Ca^2+^-intensifying EF-hand motifs (magenta). Four fragments (αN–5, α6–11, α12–16, and α17–C) were generated for expression. **(B)** His-tagged PkTRAg40.1 was incubated with GST-tagged spectrin alpha fragments, and interactions were assessed by His pull-down assays followed by western blotting with anti–6 × His and anti-GST monoclonal antibodies. PkTRAg40.1 specifically bound to α6–11 and α12–16 fragments. Arrowheads indicate protein bands (blue, PkTRAg40.1; red, α6–11 and α12–16). **(C)** GST pull-down assays using native PkTRAg40.1 from *P. knowlesi* confirmed specific interaction with the α6–11 and α12–16 fragments. GST alone was included as a negative control.

To delineate the spectrin alpha region involved in binding, the protein was divided into four overlapping fragments: αN–5 (2–580 aa), α6–11 (578–1289 aa), α12–16 (1288–1834 aa), and α17–C (1810–2419 aa) (Fig 6A and S2B). His pull-down assays demonstrated that PkTRAg40.1 interacts with both α6–11 and α12–16 fragments, showing stronger binding to α12–16, suggesting that this region is the principal binding site (Figs 6B and S9). This was further confirmed using GST-tagged spectrin fragments in pull-down assays with *P. knowlesi* late trophozoite and schizont-enriched parasite lysates. Immunoblotting with anti-PkTRAg40.1 antibodies detected native PkTRAg40.1 predominantly in the α6–11 (75–100 kDa) and α12–16 (75–100 kDa, ~ 250 kDa) fractions, whereas no reactivity was observed in the GST negative control. Notably, the strongest signal was detected in the α12–16 fraction, supporting a native interaction (Fig 6C).

**Fig 7:**
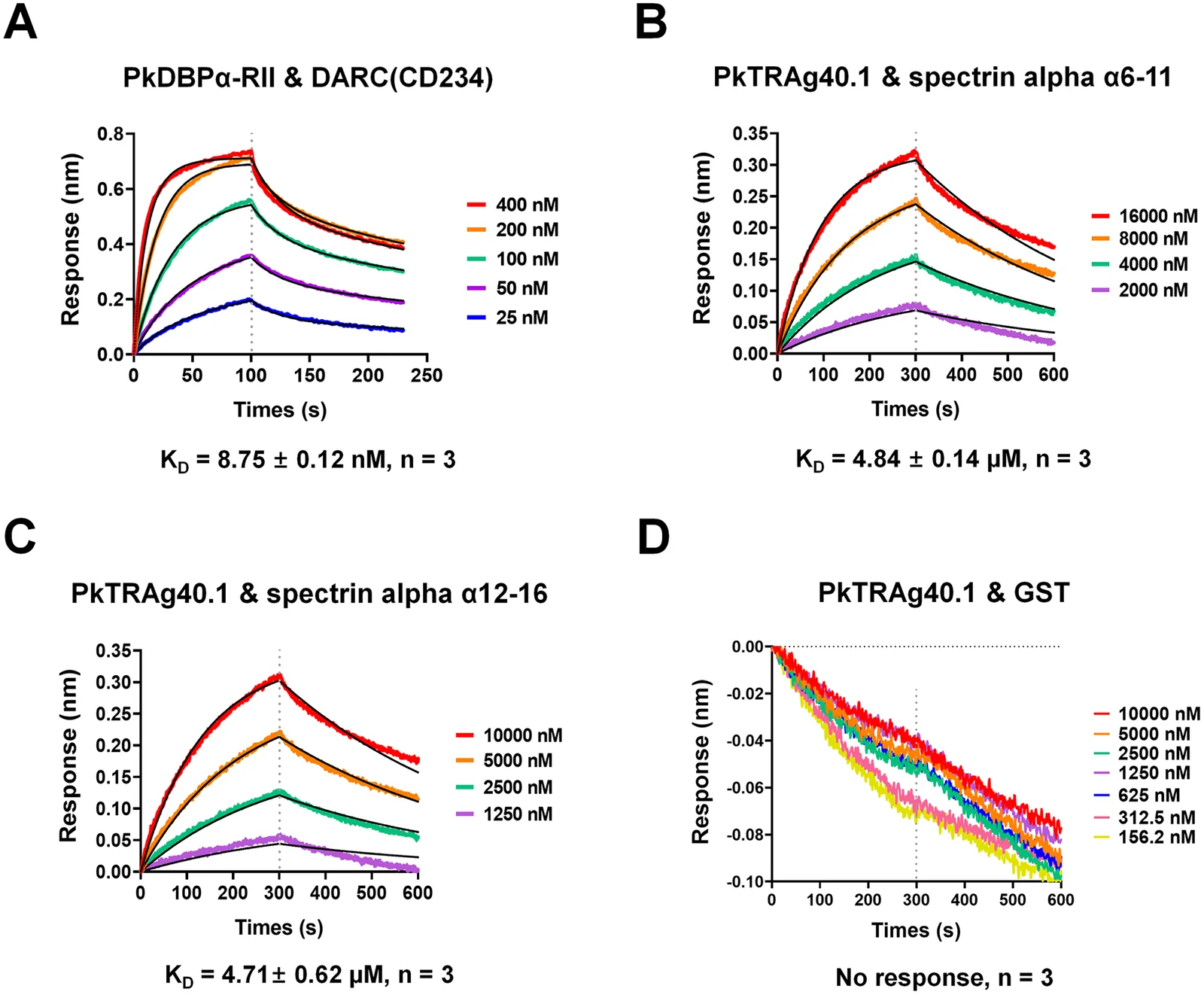
Binding affinity of PkTRAg40.1 to spectrin alpha fragments measured by biolayer interferometry. Biolayer interferometry (BLI) was used to assess the kinetics and affinity of PkTRAg40.1 binding to spectrin alpha fragments α6–11 and α12–16. Binding curves were fitted to kinetic data, and affinity constants were calculated. PkTRAg40.1 interactions with spectrin alpha followed a globally fitted 1:1 binding model, whereas the positive control interaction between PkDBPα-RII and DARC (CD234) conformed to a heterogeneous 2:1 binding model. **(A)** Binding kinetics of PkDBPα-RII with DARC (positive control). **(B, C)** Sensorgrams of PkTRAg40.1 binding to GST-tagged spectrin alpha fragments α6–11 and α12–16. **(D)** Binding profile of PkTRAg40.1 with GST alone as a negative control. All experiments were performed in triplicate, and mean values are shown.

To further characterize these interactions, binding kinetics were evaluated by bio-layer interferometry. As a positive control, PkDBPα-RII exhibited a high-affinity interaction with DARC (K_D_ = 8.75 ± 0.12 nM) (Fig 7A). PkTRAg40.1 showed specific binding to spectrin alpha fragments α6–11 and α12–16 with micromolar affinities (K_D_ = 4.84 ± 0.14 μM and 4.71 ± 0.62 μM, respectively) (Fig 7B and 7C). In contrast, no detectable binding was observed with GST, which served as the negative control (Fig 7D).

## Discussion

Tryptophan-rich antigens (TRAgs) constitute a multi-gene family conserved across *Plasmodium* species and are characterized by a C-terminal tryptophan-rich (TR) domain [19]. While *P. vivax* and *P. knowlesi* encode 36 and 26 TRAg genes, respectively, *P. falciparum* contains only three homologs known as TryThrA [19,25,36]. Although several TRAgs have been shown to bind erythrocytes and elicit strong immune responses, their molecular functions during the intraerythrocytic developmental cycle remain poorly defined [4,21,25]. Previous studies have implicated certain TRAgs in host cell remodeling and protein trafficking. For example, PvTRAg (PVP01_0000100) binds preferentially to reticulocytes and exhibits structural similarity to BAR domains, suggesting a potential role in membrane remodeling. Furthermore, it was shown to bind the negatively charged glycolipid sulfatide, a glycosphingolipid enriched in the outer leaflet of plasma membranes, supporting the idea that lipid recognition may contribute to TRAg function [19,37]. Structural modeling indicated that PkTRAg40.1 shares the elongated α-helical architecture characteristic of previously characterized TRAgs, including proteins implicated in membrane remodeling. Consistent with the emerging concept that certain TRAg family members interact with membrane lipids, recombinant PkTRAg40.1 exhibited weak but reproducible preferential reactivity toward phosphatidic acid (PA) in a lipid dot blot assay. PA is an anionic glycerophospholipid with unique biophysical properties that contribute to membrane curvature, membrane remodeling, and vesicular membrane dynamics through the recruitment and organization of membrane-associated proteins [38,39]. Together with its localization to parasite-induced membranous structures and its interaction with spectrin alpha, this observation is consistent with the membrane-associated biochemical properties of PkTRAg40.1 and raises the possibility that lipid recognition contributes to its association with parasite-induced membranous compartments. In *P. vivax*, other TRAgs have been shown to colocalize with the caveola-vesicle complex (CVC), a unique membrane structure implicated in protein trafficking [24,25]. Similarly, *P. falciparum* TryThrA colocalized with PfSBP1, which is Maurer’s cleft-associated protein, suggesting a conserved role for these proteins in host cell remodeling and parasite protein export [18,40]. These observations suggest that TRAgs have multiple functions beyond merozoite invasion. Although some have been associated with erythrocyte binding and others with protein trafficking, it remains unclear whether TRAgs fall into distinct functional classes or if individual members perform both roles. PkTRAg40.1 colocalized with PkSBP1 and exhibited a punctate distribution beneath the erythrocyte membrane, consistent with its association with SMCs, parasite-derived membranous structures analogous to the Maurer's clefts of *P. falciparum* [41,42]. These clefts are involved in the trafficking of exported parasite proteins and contribute to host cell remodeling. The localization and biochemical characteristics of PkTRAg40.1 are consistent with its association with parasite-induced membranous structures. Although PkTRAg40.1 closely colocalized with PkSBP1, it exhibited a distinct biochemical profile, being predominantly associated with detergent-resistant insoluble fractions. Despite their close colocalization, the two proteins exhibited markedly different protease accessibility profiles. In contrast to the partial protection observed for PkSBP1, PkTRAg40.1 was highly susceptible to protease digestion under all permeabilization conditions, suggesting that these proteins may occupy distinct topological positions within SMCs. Further supporting this distinct biochemical profile, sequential biochemical extraction showed that native PkTRAg40.1 remained largely resistant to hypotonic lysis, sodium carbonate, and urea extraction and was only partially solubilized by Triton X-100. This extraction profile indicates that PkTRAg40.1 is strongly associated with detergent-resistant insoluble structures rather than existing as a freely soluble parasite protein. Together with its localization to SMCs, these biochemical characteristics further support the association of PkTRAg40.1 with parasite-induced membranous compartments. However, the detergent-resistant extraction profile alone does not distinguish whether this biochemical property reflects direct membrane association, stable protein-protein interactions, incorporation into higher-order molecular complexes, or other forms of detergent-resistant protein assemblies. In addition, although higher-molecular-weight PkTRAg40.1 species were reproducibly detected by immunoblot analysis, their biochemical nature remains unresolved. Further biochemical characterization will be required to determine whether these species represent oligomeric assemblies, protein-associated complexes, or other biochemical forms of PkTRAg40.1, as well as to clarify their potential biological significance. Similar topological diversity has been described for the *P. falciparum* Maurer's cleft protein Pf332 [43]. The distinct topological profile of PkTRAg40.1, together with its interaction with erythrocyte spectrin alpha, is consistent with its localization near the host erythrocyte membrane skeleton. Several exported *P. falciparum* proteins, including KAHRP, EMP3, RESA, SBP1, and SURFIN, directly bind to spectrin, stabilizing these trafficking mechanisms and mediating host cell modifications that are critical for parasite survival [44–47]. Spectrin has also been shown to anchor Maurer’s clefts and influence their spatial distribution within infected erythrocytes [48]. The observation that PkTRAg40.1 is associated with SMCs and interacts with spectrin alpha suggests a potential link between parasite-derived membranous structures and the host erythrocyte cytoskeleton. Whether this interaction contributes to intracellular protein trafficking or host erythrocyte remodeling remains to be determined. Collectively, reports describing interactions between *Plasmodium* proteins and spectrin alpha raise the possibility that host genetic variation in SPTA1 may influence parasite biology and malaria susceptibility. Although exported proteins from *P. falciparum* provide a useful conceptual framework for interpreting these findings, *P. knowlesi* differs biologically from other human malaria parasites, particularly in its erythrocyte tropism. Therefore, the spectrin alpha interaction identified for PkTRAg40.1 should be interpreted as a species-specific finding, and whether analogous mechanisms operate in other *Plasmodium* species remains to be established.

In this study, we characterized PkTRAg40.1 and identified spectrin alpha (*SPTA1*) as its host binding partner. Spectrin, a principal component of the erythrocyte cytoskeleton, forms an extended meshwork beneath the plasma membrane in association with spectrin beta, actin, ankyrin, and protein 4.1R [49]. This network provides the erythrocyte with the mechanical resilience and elasticity required for circulation through narrow capillaries [50]. Mutations in *SPTA1* that disrupt the erythrocyte cytoskeleton cause hereditary spherocytosis (HS) and elliptocytosis (HE), emphasizing the essential role of spectrin in preserving membrane integrity and cellular deformability [51]. PkTRAg40.1 bound specifically to the α6–11 and α12–16 regions of erythrocyte spectrin alpha, with stronger interaction observed for α12–16, identifying the C-terminal region of spectrin alpha as its principal binding site. Given the structural role of spectrin alpha in the erythrocyte membrane skeleton, this interaction may contribute to the stabilization of parasite-derived membranous compartments. Similarly, spectrin alpha exhibits domain-specific binding properties, as demonstrated by the interaction of the PfKAHRP K2 domain with the α12–16 fragment and PfEMP3 with the α17–C region [46,47]. These interactions are thought to modulate the host cell membrane, either by stabilizing parasite-induced structures or contributing to membrane destabilization, depending on the specific protein involved [46,52]. PkTRAg40.1 binds spectrin alpha with micromolar affinity, similar to other exported parasite proteins that target host cytoskeletal components [35,46].

The association of PkTRAg40.1 with SMCs, together with its interaction with spectrin alpha, is consistent with a role in the organization of parasite-induced membranous compartments and their association with the host erythrocyte cytoskeleton. Given its localization pattern, its similarity to cleft-associated proteins in *P. falciparum* and *P. knowlesi*, its interaction with erythrocyte spectrin alpha, and its membrane-associated biochemical properties, PkTRAg40.1 may contribute to the structural organization of parasite-derived membranous compartments within infected erythrocytes. Although the precise biological role of PkTRAg40.1 remains to be determined, gene essentiality data from *P. knowlesi* PiggyBac screening indicate that it is non-essential *in vitro*, suggesting that its function may be conditionally important *in vivo* [53]. Immunofluorescence analyses demonstrated the presence of PkTRAg40.1 throughout the intraerythrocytic developmental cycle, but stage-specific changes in protein abundance were not quantitatively assessed. Consequently, whether differential expression contributes to the biological function or immunogenicity of PkTRAg40.1 remains to be determined. Given its interaction with erythrocyte spectrin alpha, PkTRAg40.1 may participate in processes associated with host cell structural organization. However, whether this interaction influences erythrocyte mechanical properties or parasite biology remains to be determined [54]. PkTRAg40.1 was recognized by IgG antibodies in 50% of patients with *P. knowlesi* malaria, demonstrating naturally acquired immune recognition but also indicating limited sensitivity for serological detection. Because the immunogenicity of individual *P. knowlesi* TRAgs remains poorly characterized, studies of the TRAg family in the phylogenetically related species *P. vivax* provide useful comparative context. Previous studies have likewise demonstrated substantial heterogeneity in naturally acquired antibody responses among individual PvTRAg family members, suggesting that variable immune recognition is a common feature of the TRAg family. The heterogeneous recognition of PkTRAg40.1 may reflect differences in stage-specific expression, antigen accessibility, parasite burden, previous malaria exposure, host immune status, and potentially genetic polymorphism, although the sequence diversity of PkTRAg40.1 has not yet been comprehensively characterized. In addition, the observed cross-reactivity with *P. vivax* sera represents an important limitation for species-specific serodiagnosis in co-endemic regions. This is likely attributable to the high conservation between PkTRAg40.1 and its *P. vivax* orthologue, including approximately 83% amino acid identity within the tryptophan-rich domain, highly similar predicted structures, and conserved predicted B-cell epitopes (S1D and S7C Figs). Collectively, these findings indicate that although PkTRAg40.1 is naturally immunogenic, its moderate sensitivity and cross-reactivity limit its utility as a stand-alone species-specific serological marker and reduce its potential as an individual vaccine antigen. Accordingly, the primary significance of the present findings lies in improving our understanding of parasite biology and parasite-induced host cell modification rather than in supporting the development of a species-specific diagnostic or vaccine antigen. While protein export and host cell remodeling have been extensively studied in *P. falciparum*, these processes remain poorly understood in non-*Laverania* species. Our findings identify spectrin alpha as a host binding partner of PkTRAg40.1 and demonstrate its association with SMCs. However, because the present study is based primarily on *in vitro* binding assays and subcellular localization, the physiological significance of this interaction remains to be determined. Future functional studies, including targeted genetic manipulation, will be required to determine whether PkTRAg40.1 plays a causal role in host cell remodeling, intracellular protein trafficking, or parasite survival *in vivo*.

## Supporting information

S1 Fig Homology-based predicted three-dimensional structures of the TRAg tryptophan-rich domain from *P. vivax* and *P. knowlesi.* **(A)** AlphaFold2-predicted structure of PkTRAg40.1 colored according to pLDDT confidence scores. Most residues within the predicted tryptophan-rich domain (TRD) exhibited high confidence scores, supporting the reliability of the structural model. **(B)** Electrostatic surface potential analysis of PkTRAg40.1. Electrostatic potential analysis of the predicted PkTRAg40.1 structure identifies positively charged patches at the N-terminal end and along the concave surface of the protein. In contrast, the C-terminal region exhibits a relatively more negatively charged surface. The structure is shown in front, 180° rotated, and side views. Blue and red indicate positive and negative electrostatic potentials, respectively. **(C)** Structural comparison of PkTRAg40.1 with previously characterized PvTRAg proteins. The TRD regions of the TRAg proteins were modeled using AlphaFold2, except for PVP01_0000100, whose structure was experimentally determined (PDB ID: 8ARL). All predicted models were subsequently refined using Galaxy Refine to improve structural accuracy. Structural alignments were performed using the DALI server, and the resulting RMSD and Z-score values are summarized in the table. **(D)** Multiple sequence alignment of the tryptophan-rich domain of PKA1H_050021800 and PVP01_0532700. Red and sky-blue bars indicate the highly conserved and less conserved amino acid residues, respectively. The 25 conserved tryptophan residues are highlighted in yellow.
(TIF)

S2 Fig SDS-PAGE analysis of recombinant proteins used in PkTRAg40.1 studies. Purified recombinant proteins tagged with hexahistidine, GST, or the Fc region of human IgG1 were separated by SDS-PAGE and stained with Coomassie blue. **(A)** Hexahistidine-tagged proteins purified using Ni-NTA resin. Lane 1, PkDBPα-RII; Lane 2, MBP. **(B)** GST-tagged proteins purified using Glutathione Sepharose 4B resin. Lane 1, PkMSP1–19 (merozoite surface protein 1); Lane 2, PkSBP1 (skeleton-binding protein 1); Lane 3, GST; Lane 4, spectrin alpha αN–5 fragment; Lane 5, α6–11 fragment; Lane 6, α12–16 fragment; Lane 7, α17–C fragment. PkMSP1–19 was used as a reference antigen in the protein microarray, and PkSBP1 was used for polyclonal antibody production in rabbits. **(C)** Human DARC (CD234) fused to hIgG1 Fc and purified using Protein G resin. Lane 1, DARC. Lane M, molecular weight marker (kDa).
(TIF)

S3 Fig Uncropped immunoblots of the PkTRAg40.1 protein solubility analysis. (TIF)

S4 Fig Uncropped immunoblots of the PkTRAg40.1 protease protection assay. (TIF)

S5 Fig Lipid dot blot analysis of recombinant PkTRAg40.1. Purified His-tagged PkTRAg40.1 and His-tagged MBP (1 μg each) were incubated with PIP Strip membranes, and bound proteins were detected using an anti-His monoclonal antibody (1:2,000) followed by IRDye 800CW goat anti-mouse IgG (1:10,000). The spot numbers and membrane locations are indicated below the blot. A detectable signal was observed for phosphatidic acid (PA; spot 14, purple circle) in the PkTRAg40.1 sample, whereas no corresponding signal was detected in the MBP control. The image shown is representative of three independent experiments.
(TIF)

S6 Fig Subcellular localization of PkTRAg40.1 in *P. knowlesi*–infected erythrocytes. **(A–C)** Immunofluorescence assays of schizont-stage parasites using mouse anti–PkTRAg40.1 serum (green) co-labeled with rabbit antibodies against PkMSP1–19 (merozoite surface), PkDBPα (microneme), or PkRAMA (rhoptry bulb) (red). Parasite nuclei were stained with DAPI (blue). **(D)** Localization throughout the asexual stages was assessed using anti–PkTRAg40.1 serum with Alexa Fluor 488–conjugated secondary antibody (green) and rabbit anti–spectrin alpha antibody with Alexa Fluor 594–conjugated secondary antibody (red). PkTRAg40.1 was predominantly detected adjacent to the erythrocyte membrane during parasite development.
(TIF)

S7 Fig Serological reactivity and predicted epitope architecture of PkTRAg40.1. **(A)** Protein microarray analysis of total IgG responses against recombinant PkMSP1–19, PkSBP1, and PkTRAg40.1 using sera from *P. knowlesi*-infected patients (n = 70) and uninfected healthy controls (n = 42). Bars indicate mean fluorescence intensity (MFI) ± standard deviation (SD). Statistical significance was assessed using Student’s *t*-test. **(B)** Receiver operating characteristic (ROC) curve analysis of PkTRAg40.1 for discrimination of *P. knowlesi*- and *P. vivax*-infected patients from healthy controls. The corresponding area under the curve (AUC) values and 95% confidence intervals are indicated. **(C)** Comparison of predicted linear and conformational B-cell epitopes between PkTRAg40.1 (PKA1H_050021800) and its *P. vivax* orthologue, TRAg (PVP01_0532700). Epitope prediction was performed using the conserved TR domain corresponding to the recombinant protein used in this study. Predicted epitopes from four independent algorithms (IEDB Emini Surface Accessibility, DiscoTope 3.0, ElliPro linear and ElliPro conformational) are displayed as horizontal bars aligned with the protein sequence. The lower panels summarize residue-wise epitope prediction frequencies, where each bar represents an individual amino acid residue and the color indicates the number of algorithms predicting that residue as part of a B-cell epitope. Colored boxes denote consensus epitope regions identified by at least two prediction methods. The upper panels show the predicted B-cell epitopes mapped onto the AlphaFold2-predicted structural models as surface representations. Residues predicted by at least two methods are highlighted as consensus epitopes (orange, ≥ 2 methods; light blue, ≥ 3 methods; red, all four methods). The red asterisk indicates an identical sequence region shared between PkTRAg40.1 and its *P. vivax* orthologue, corresponding to a conserved predicted B-cell epitope that could potentially contribute to the observed cross-reactive antibody responses.
(TIF)

S8 Fig GST negative control used in the pull-down assay with erythrocyte membrane proteins. Purified GST and GST-fusion proteins used in the pull-down assays were analyzed by SDS–PAGE followed by silver staining. GST was included as a negative control to evaluate non-specific interactions with erythrocyte membrane proteins. Lane M, molecular weight marker; Lane 1, GST; Lane 2, erythrocyte membrane proteins incubated with GST.
(TIF)

S9 Fig SDS-PAGE analysis of the His pull-down assay for PkTRAg40.1 and spectrin alpha fragments. His-tagged PkTRAg40.1 was incubated with four recombinant GST-tagged spectrin alpha fragments. Eluates from the His pull-down assays were resolved by SDS-PAGE and stained with Coomassie blue to assess protein interactions. PkTRAg40.1 showed specific binding to the α6–11 and α12–16 fragments. Arrows indicate protein bands corresponding to α6–11 and α12–16 (red) and PkTRAg40.1 (blue).
(TIF)

S1 Table Primer sequences for recombinant protein expression. (DOCX)

S2 Table Peptide sequences of spectrin alpha and hemoglobin subunit beta identified by MALDI-TOF/MS analysis. (DOCX)

S1 File Raw Gel. Original uncropped gel and immunoblot images. This file contains the original uncropped Coomassie-stained SDS-PAGE, silver-stained gel, and immunoblot images underlying Figs 1, 2, 5, 6, S2, S8, and S9. Red dashed boxes indicate the regions shown in the corresponding figures.
(PDF)
