# Supplementary material for: Plasmodium knowlesi tryptophan-rich antigen 40.1 binds human erythrocyte spectrin alpha
Source: PLoS Negl Trop Dis. 2026 Sep 17;20(9):e0014700. doi: 10.1371/journal.pntd.0014700 (PMC13585231; doi:10.1371/journal.pntd.0014700)
Supplement: Supplementary file 1 [file pntd.0014700.s012.pdf]

F1_raw_images

C

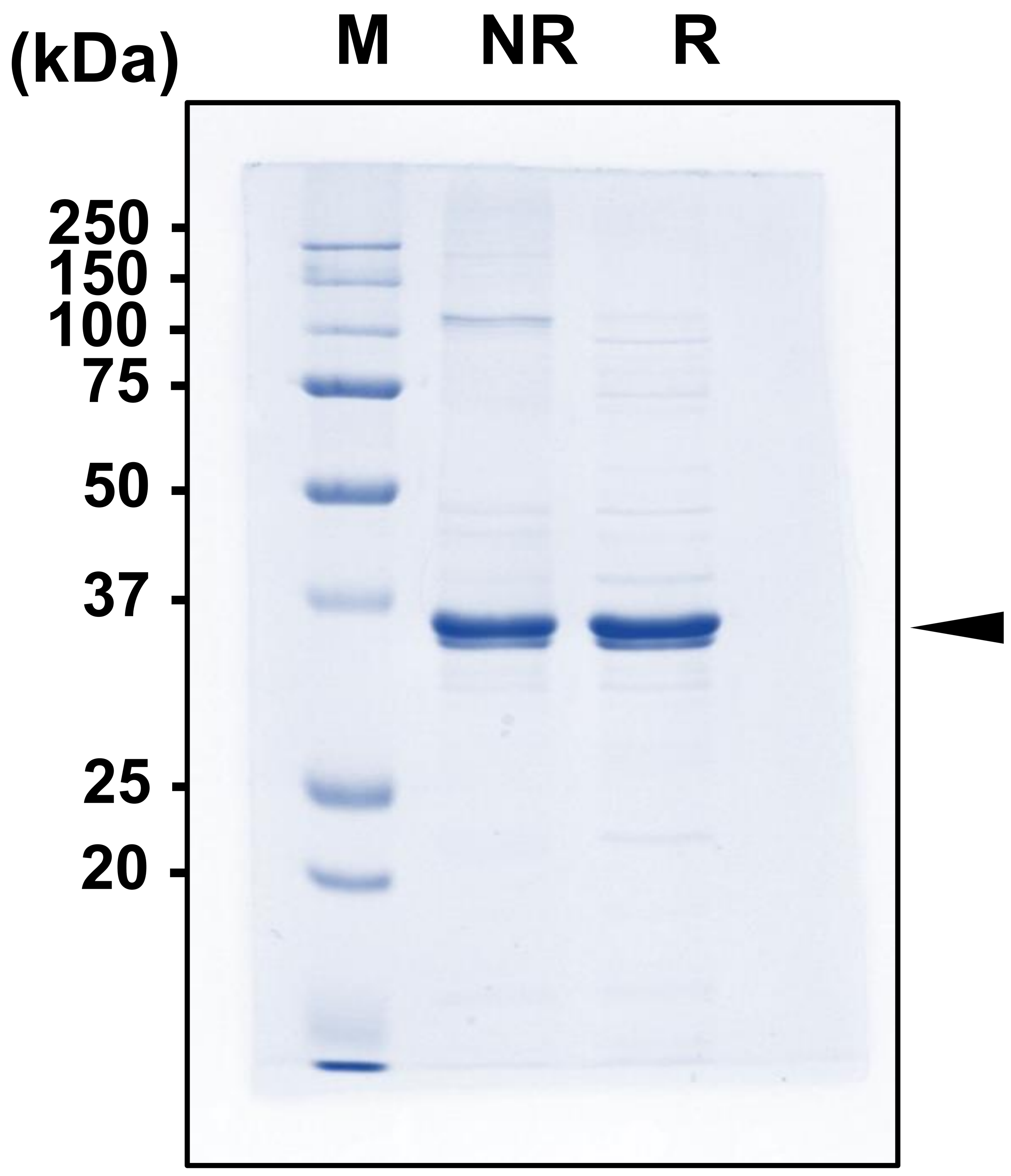

D

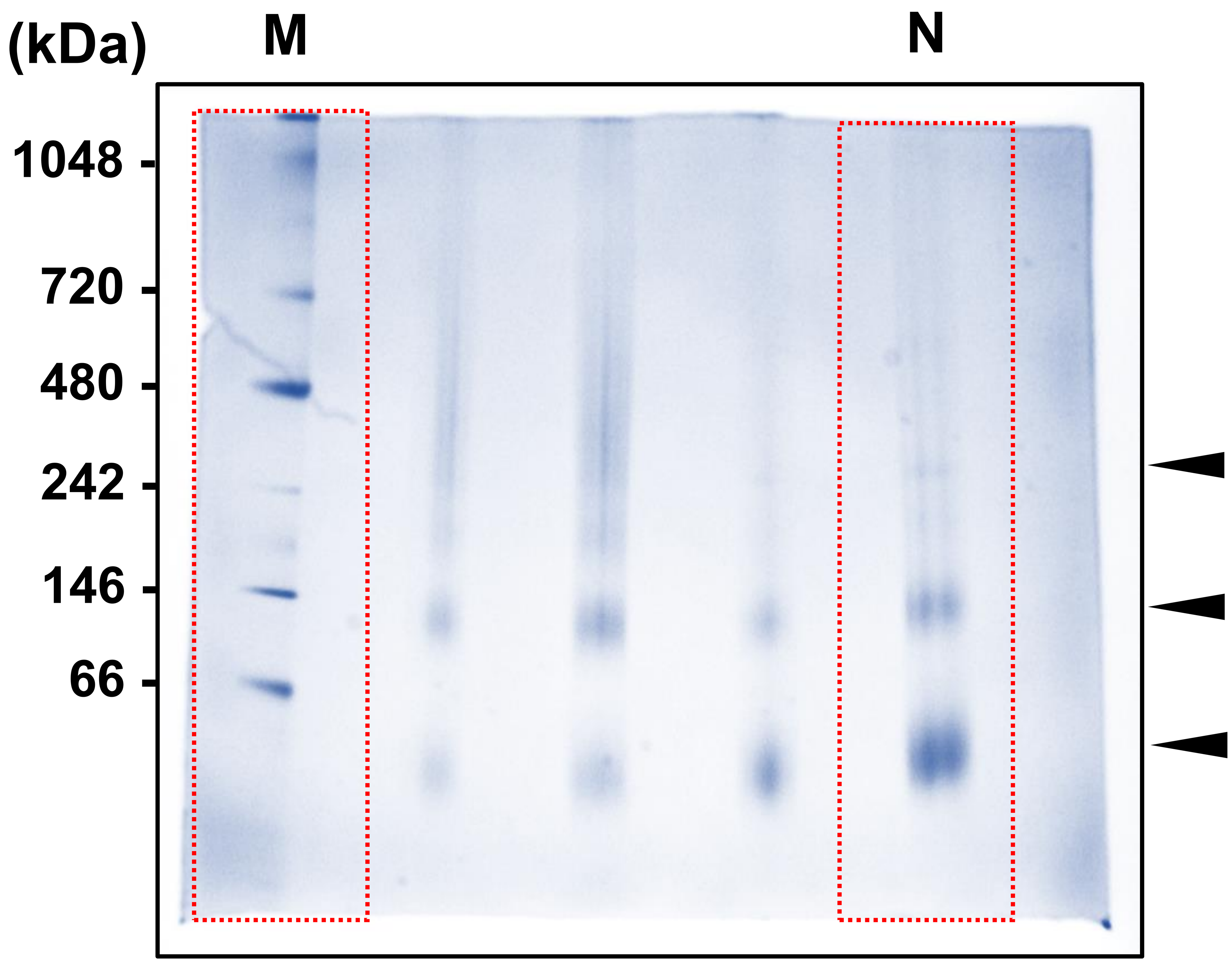

E

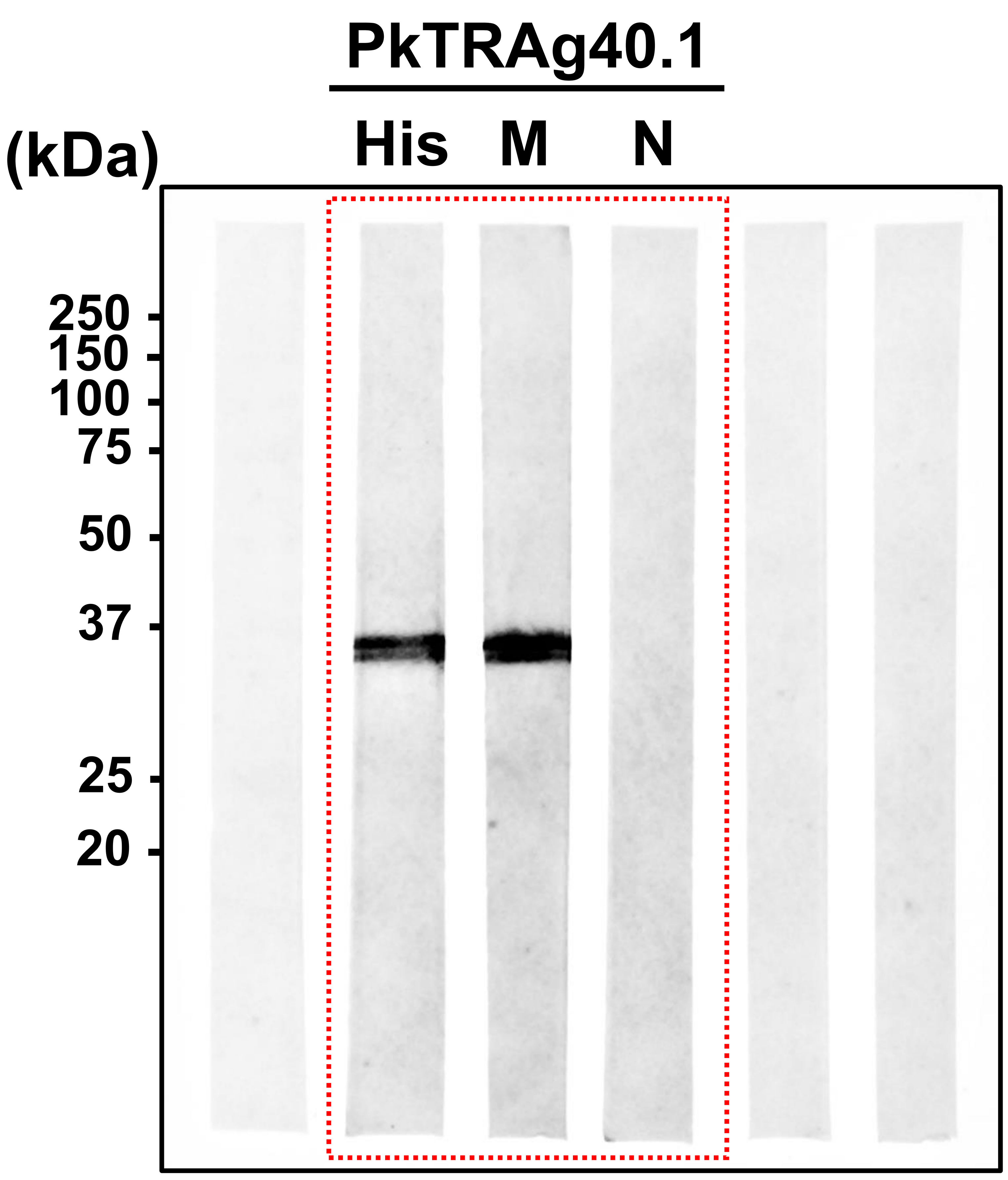

F1C–E raw images. Original uncropped Coomassie-stained SDS-PAGE images underlying Fig 1C–E. The red dashed boxes indicate the regions shown in Fig 1C-E.

F2A_raw_images

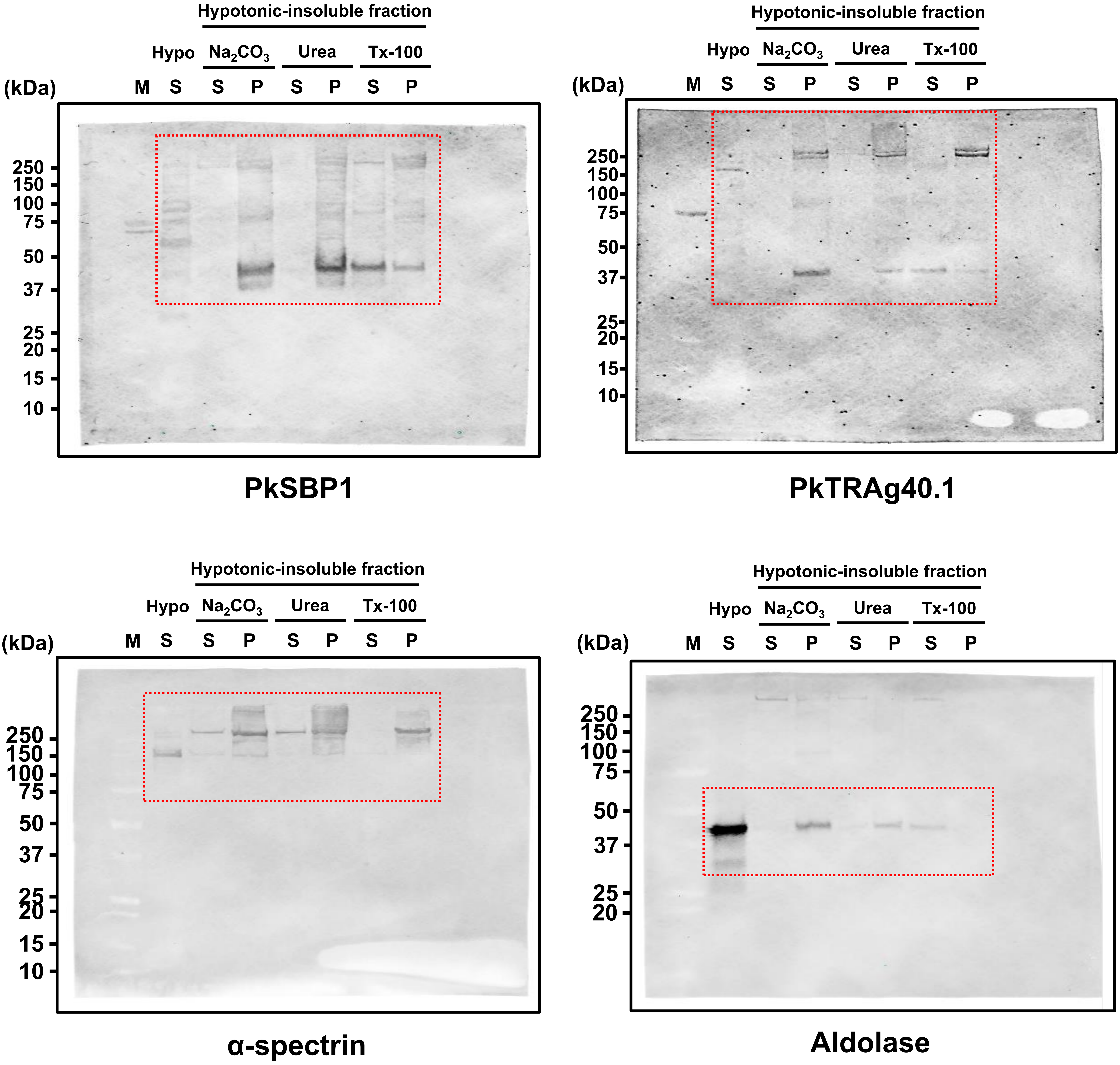

Fig 2A raw images. Original uncropped western blot images underlying Fig 2A.
The red dashed boxes indicate the regions shown in Fig 2A.

F2B_raw_images

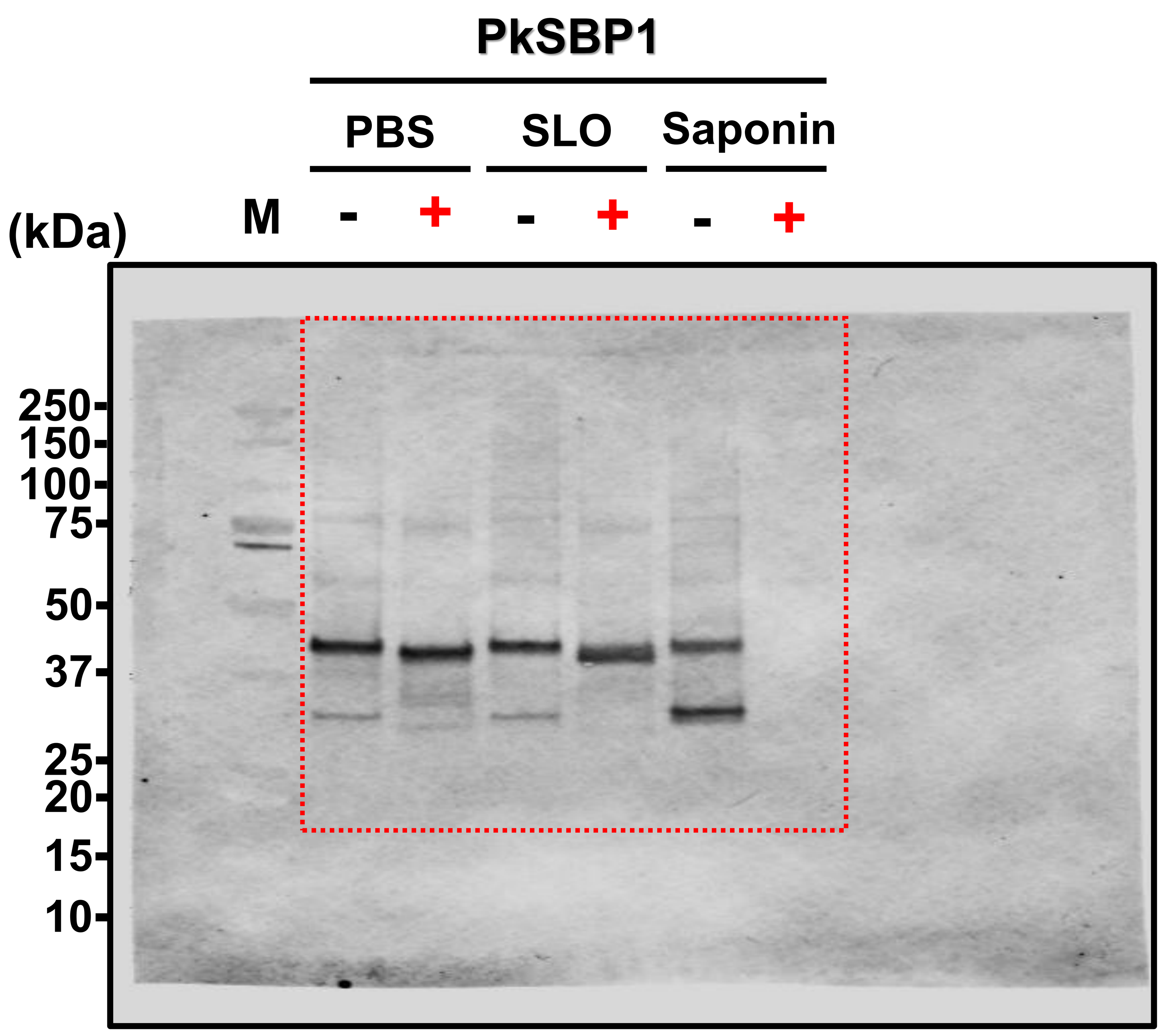

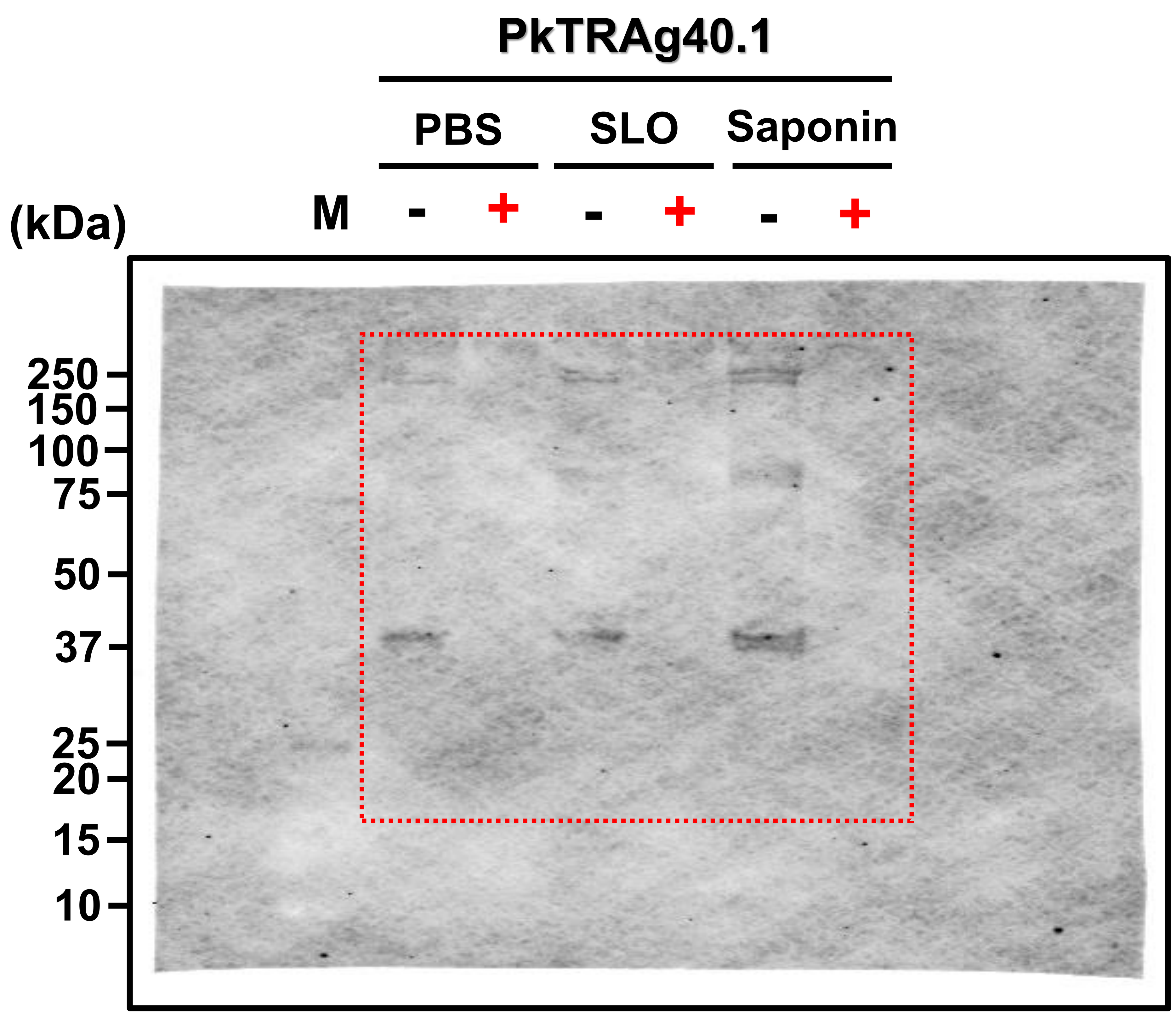

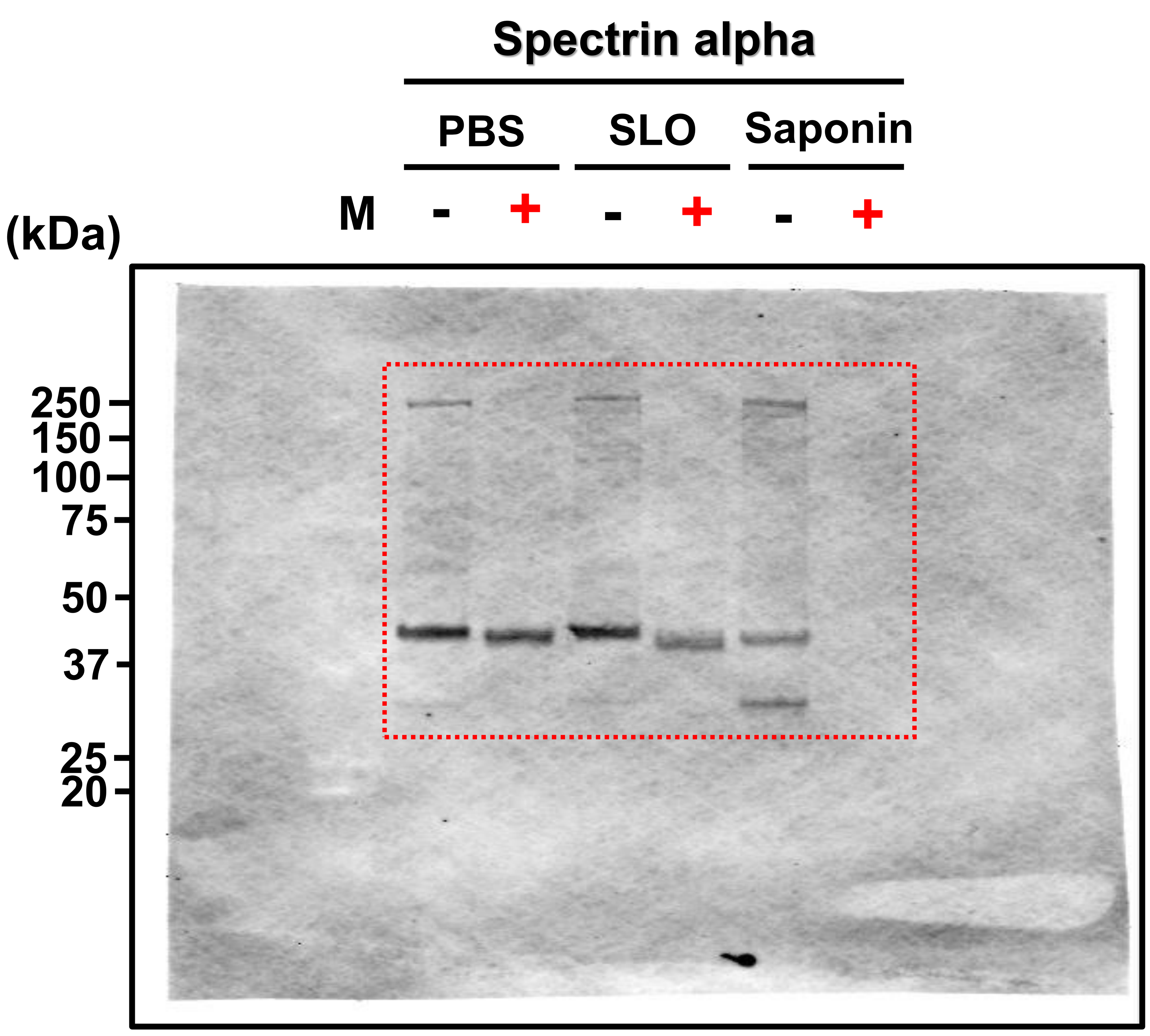

Fig 2B raw images. Original uncropped western blots images underlying Fig 2B. The red dashed boxes indicate the regions shown in Fig 2B.

F5_raw_images

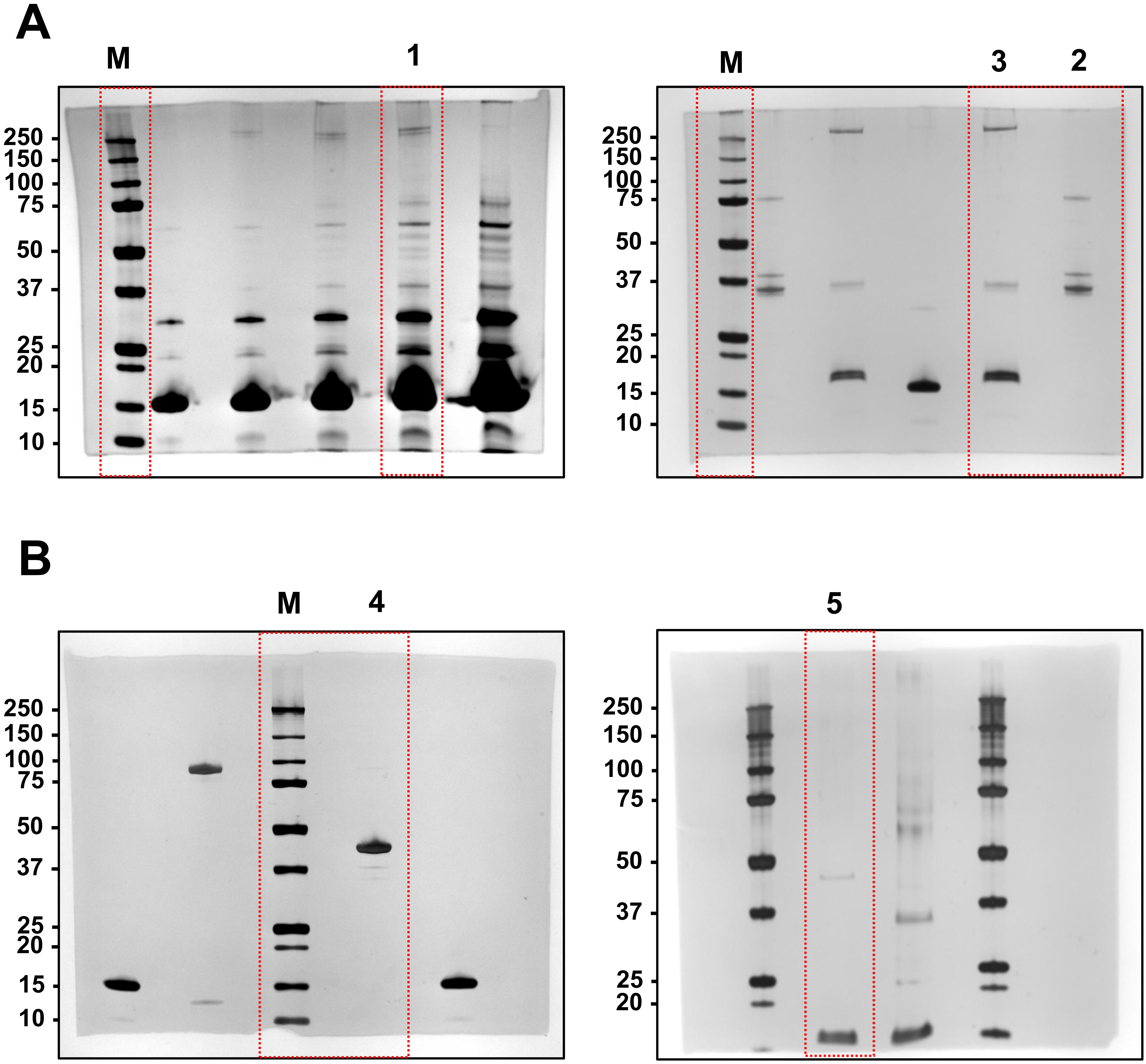

Fig 5 raw images. Original uncropped silver-stained gel images underlying Fig 5A and 5B. The red dashed boxes indicate the regions shown in Fig 5.

F6_raw_images

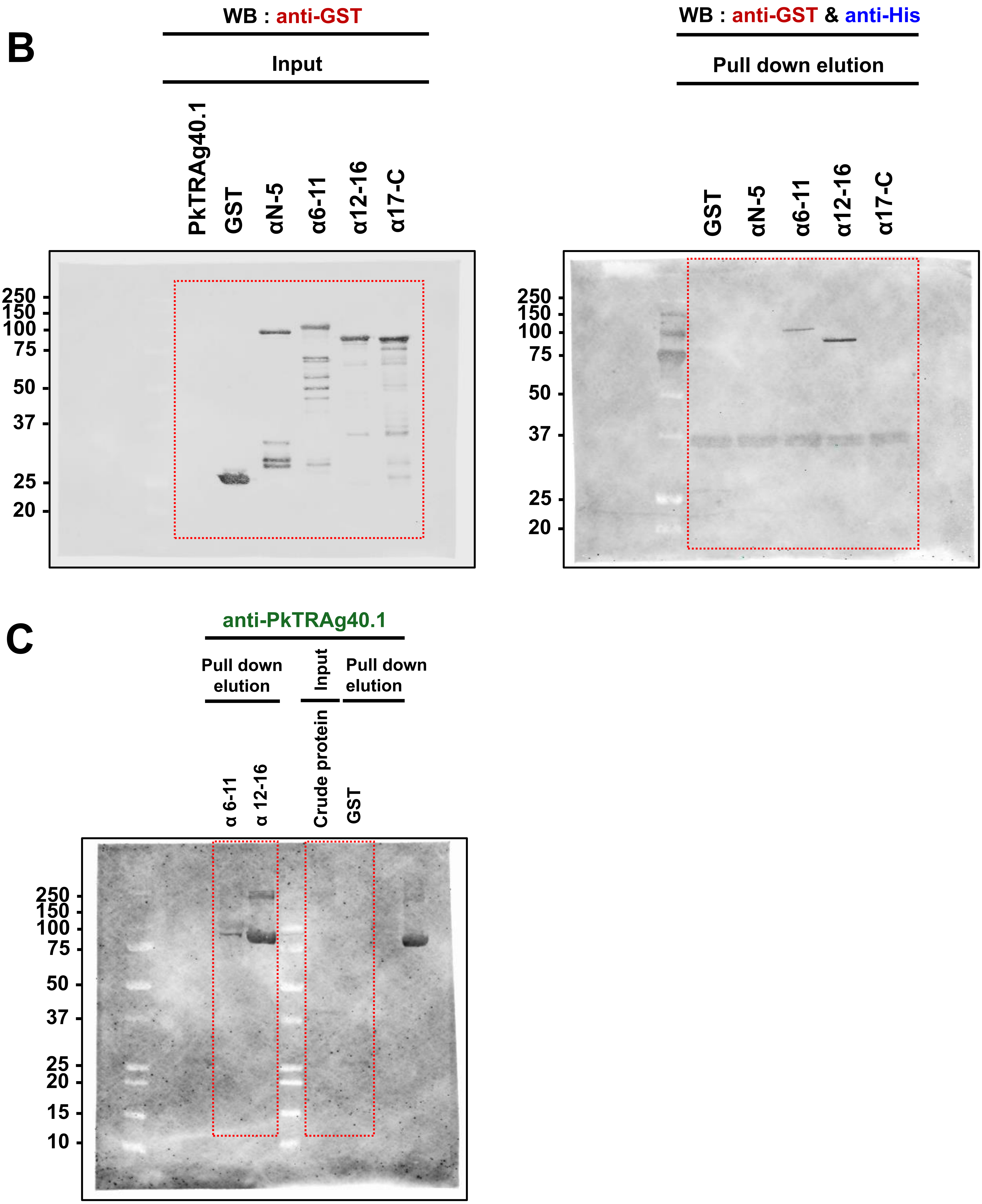

Fig 6B–C raw images. Original uncropped western blot underlying Fig 6B and 6C. The red dashed boxes indicate the regions shown in Fig 6.

S2_raw_images

A

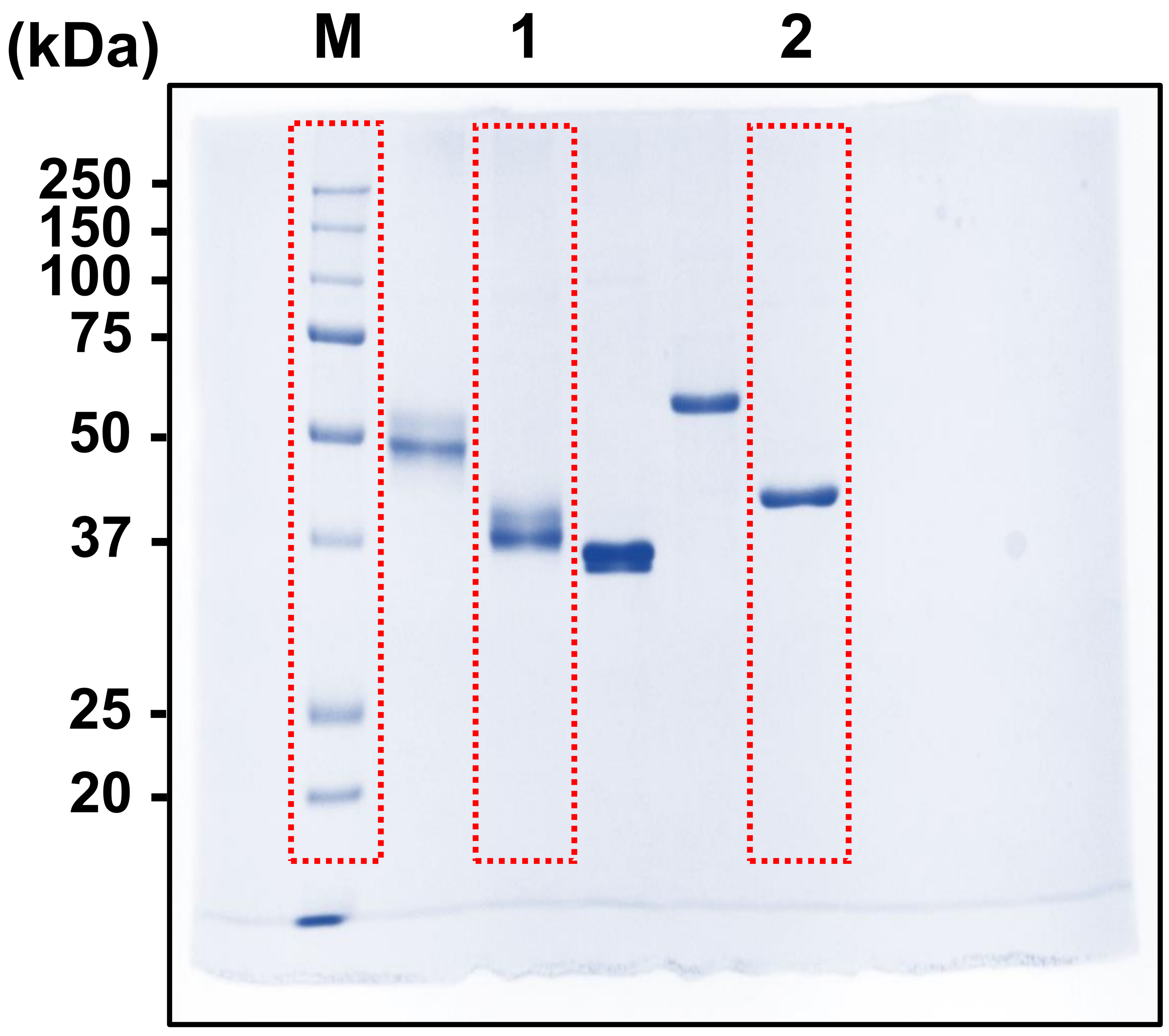

B

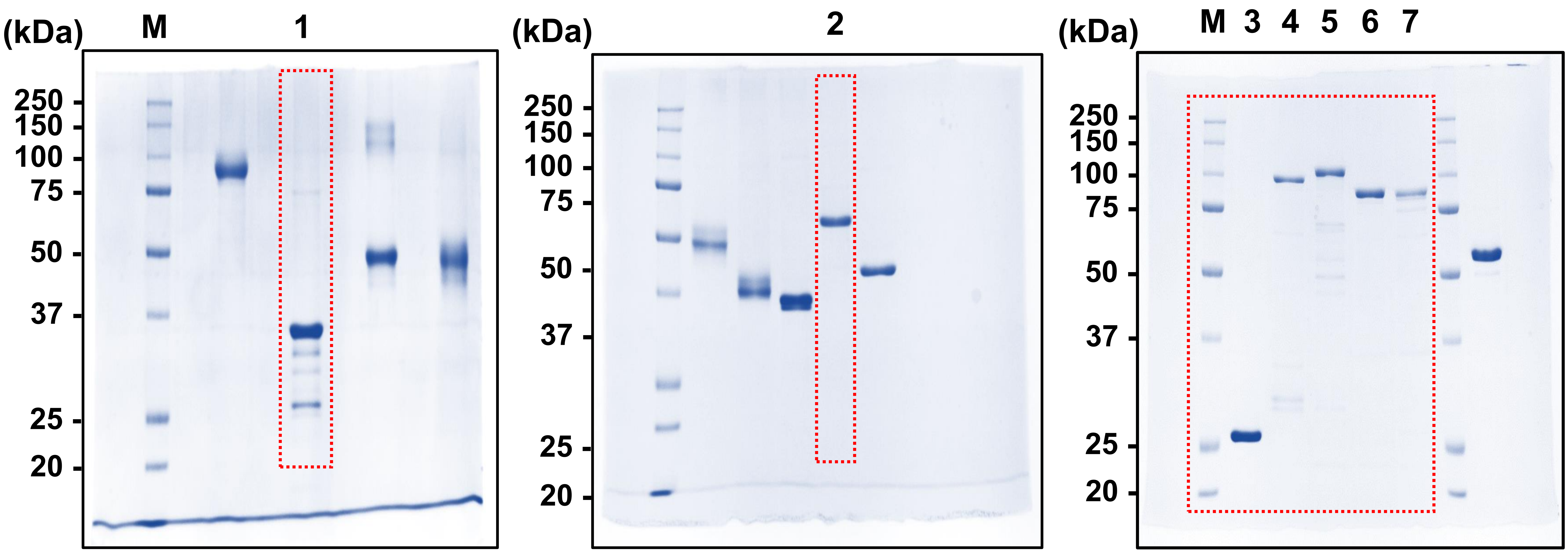

C

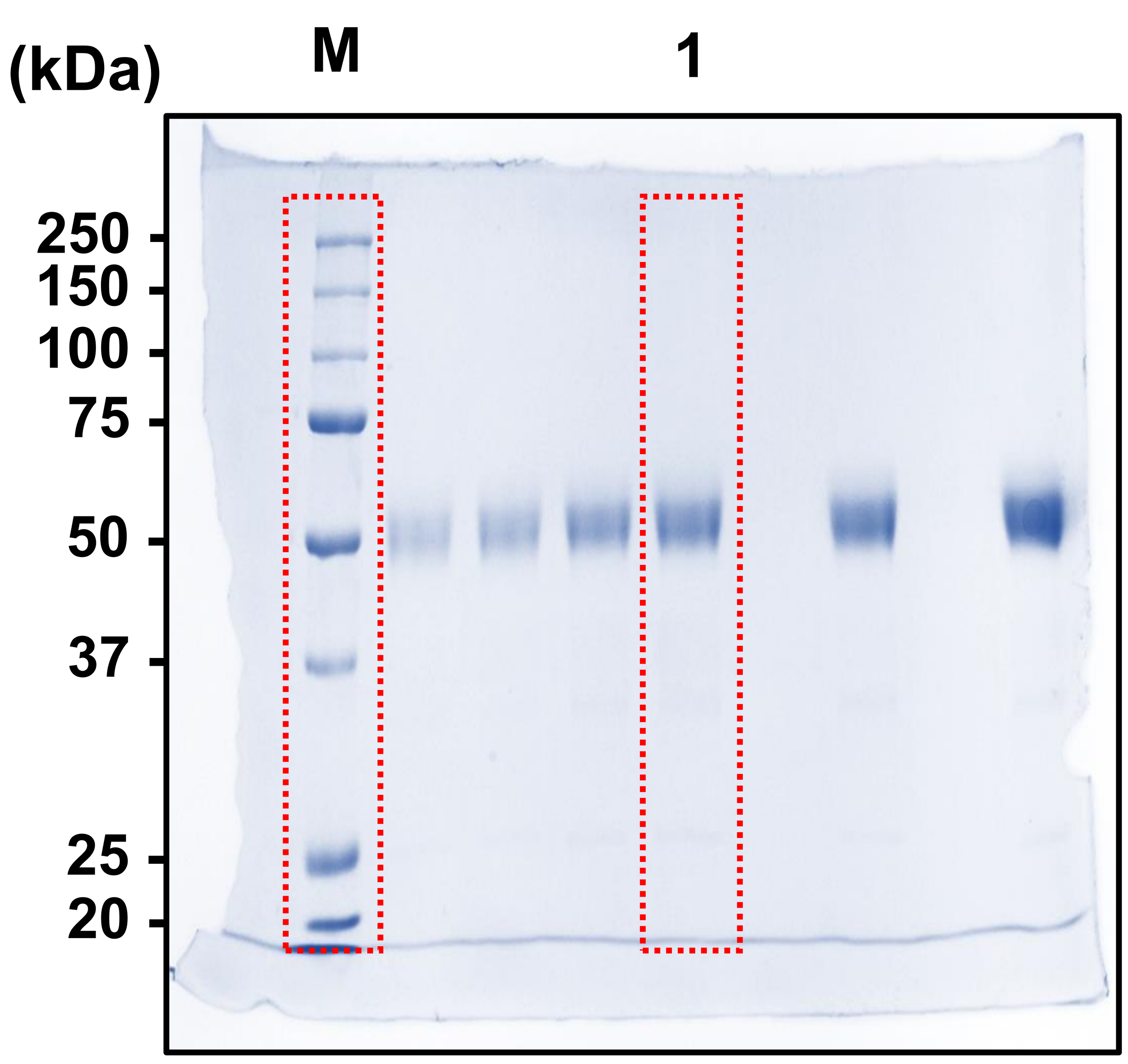

S2 raw images. Original uncropped Coomassie-stained SDS-PAGE images underlying S2A-C Fig.
The red dashed boxes indicate the regions shown in S2A-C Fig.

S8_raw_image

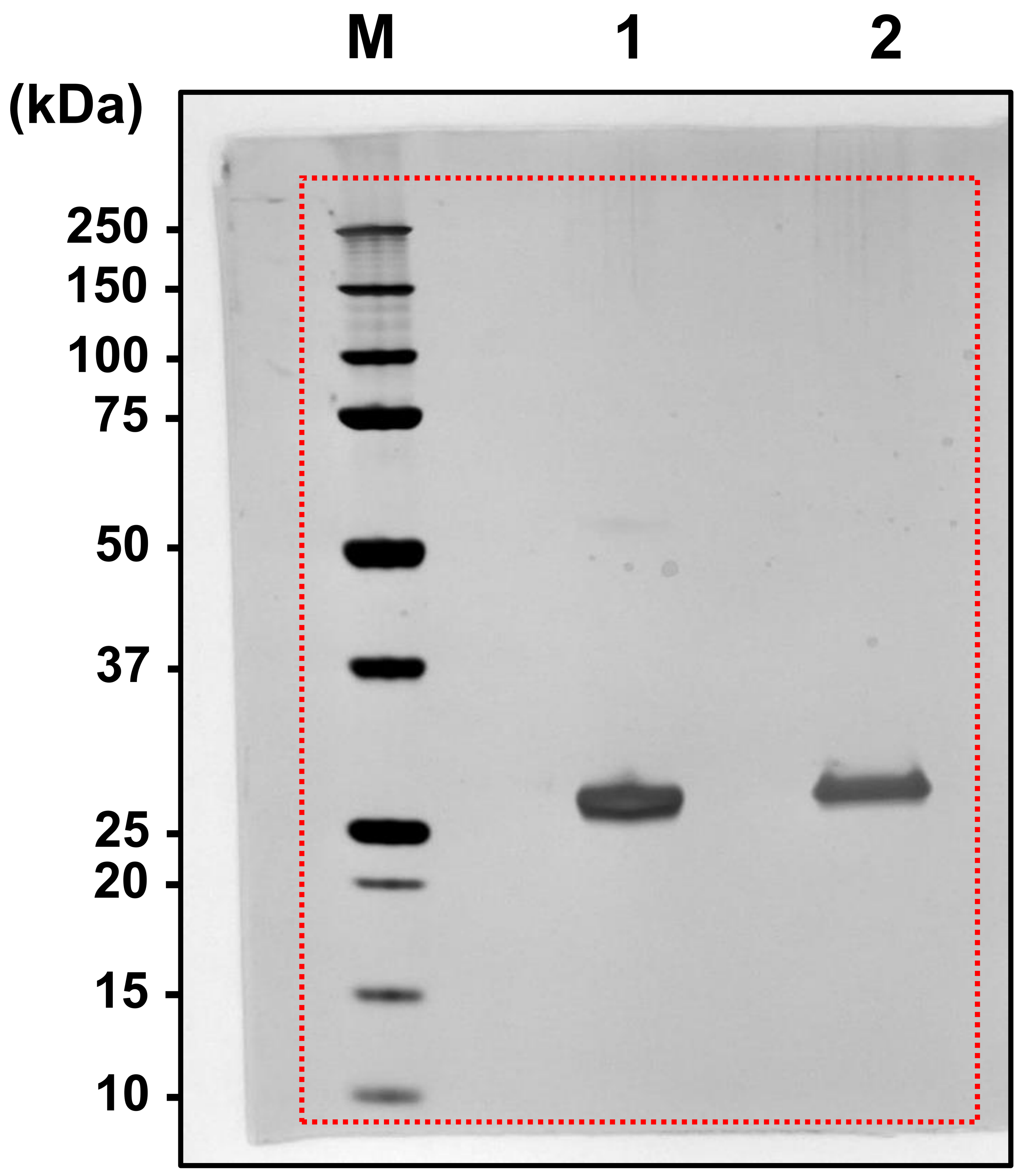

S8 raw image. Original uncropped silver-stained gel underlying S8 Fig.
The red dashed boxes indicate the regions shown in S8 Fig.

S9_raw_image

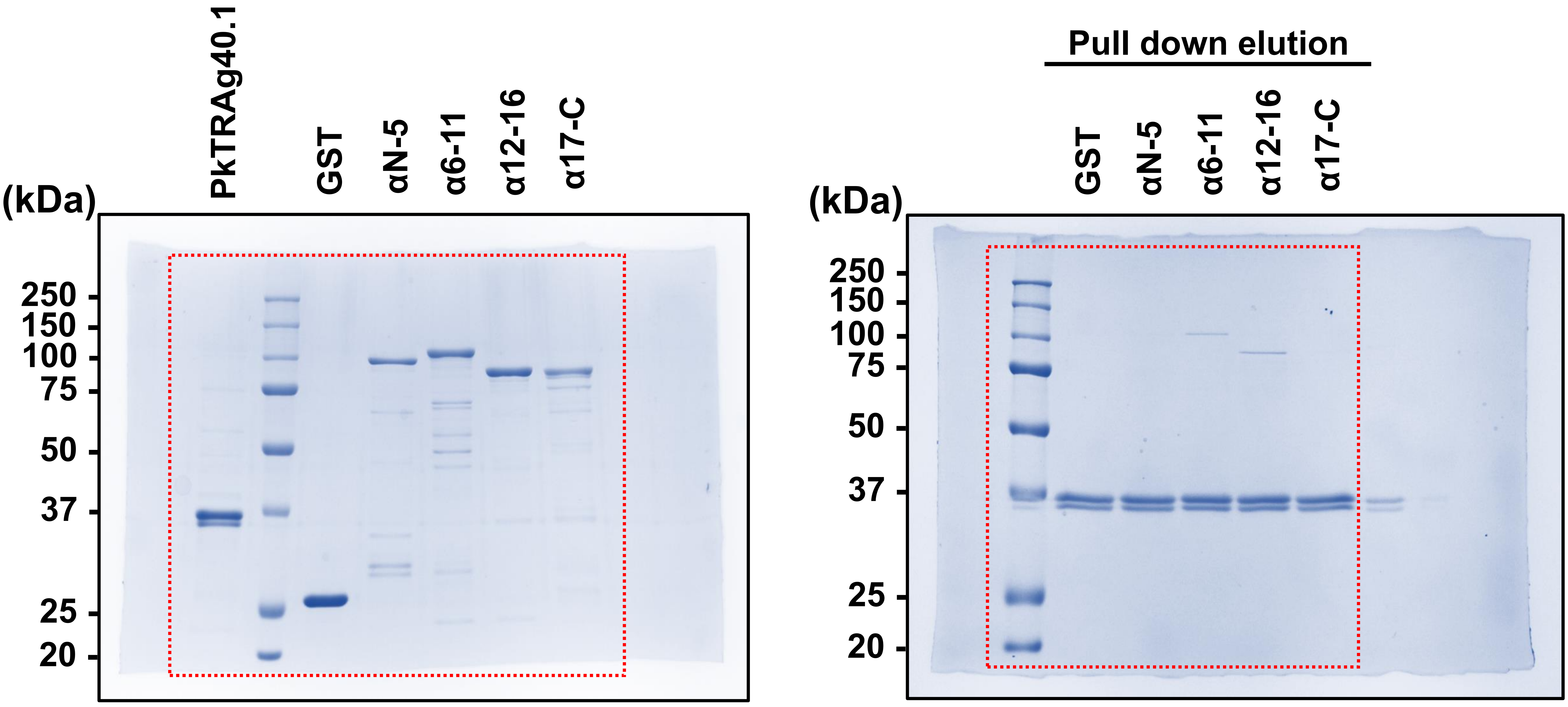

S9 raw image. Original uncropped Coomassie-stained SDS-PAGE underlying S9 Fig. The red dashed boxes indicate the regions shown in S9 Fig.
