# Supplementary material for: Plasmodium knowlesi tryptophan-rich antigen 40.1 binds human erythrocyte spectrin alpha
Source: PLoS Negl Trop Dis. 2026 Sep 17;20(9):e0014700. doi: 10.1371/journal.pntd.0014700 (PMC13585231; doi:10.1371/journal.pntd.0014700)
Supplement: Supplementary file 2 [file pntd.0014700.s010.docx]

**S1 Table. Primer sequences for recombinant protein expression.**

| **List of primers for *E. coli* recombinant proteins expression** | | | | |
| --- | --- | --- | --- | --- |
| **Target** | **Forward and reverse primer (5’→3’)** | **Vector** | **Position**  **(aa.)** | **MW**  **(kDa)** |
| PkTRAg40.1_F | GGCGCCATGGGATCC GAGGTAGATAAGACCTCA | pProEX-HTB | 67-325 | 35.9 |
| PkTRAg40.1_R | ACCGCATGCCTCGAG TCAACTGATGAACATAGATGCTGGAG | pProEX-HTB |  |  |
| PkSBP1_F | GTTCCGCGTGGATCC ACCGCTACCACTACCACTGT | pGEX4T-2 | 38-183 | 41.8 |
| PkSBP1_R | ATGCGGCCGCTCGAGTCA GGATGGGCTGATGTCGA | pGEX4T-2 |  |  |
| SPTA1_α N-5_F | GTTCCGCGTGGATCC GAGCAATTTCCAAAGGAAACCGTT | pGEX4T-1 | 2-580 | 94.0 |
| SPTA1_α N-5_R | CACGATGCGGCCGCA TCACAATGACTCCTTCAGCAATCTAC | pGEX4T-1 |  |  |
| SPTA1_α 6-11_F | GTTCCGCGTGGATCC CTGAAGGAGTCATTGCTTCTGCA | pGEX4T-1 | 578-1289 | 108.5 |
| SPTA1_α 6-11_R | CACGATGCGGCCGCA TCACTGGGCCTCATTTAGGCT | pGEX4T-1 |  |  |
| SPTA1_α 12-16_F | GTTCCGCGTGGATCC GCCCAGAAATTCTACCTGTTCCTC | pGEX4T-1 | 1288-1834 | 89.8 |
| SPTA1_α 12-16_R | CACGATGCGGCCGCA TCACCAAGCTTCCTCTTCCTCAG | pGEX4T-1 |  |  |
| SPTA1_α 17-C_F | GTTCCGCGTGGATCC GCCCGAGGACTTAAGTTGGAAG | pGEX4T-1 | 1810-2419 | 96.6 |
| SPTA1_α 17-C_R | CACGATGCGGCCGCA TCAGTTGCCAAAGTAGGAATTGGTG | pGEX4T-1 |  |  |
| MBP_F | GGCGCCATGGGATCC AATCGAAGAAGGTAAACTGGTAATCTG | pProEX-HTB | 27-392 | 44.8 |
| MBP_R | ACCGCATGCCTCGAG AGTCTGCGCGTCTTTCAGG | pProEX-HTB |  |  |
| **List of primers for HEK293 recombinant proteins expression** | | | | |
| **Target** | **Forward and reverse primer (5’→3’)** | **Vector** | **Position**  **(aa.)** | **MW**  **(kDa)** |
| PkDBPα-RII_F | CTTGGA GCGGCCGCAAGTGGGCACCCCGACGGAATTA | pTT5 | 155-518 | 45.4 |
| PkDBPα-RII_R | TACAGGTTCTC TGGCGCGCCCTTGACGTTCTCTTGCGTG |  |  |  |
| CD234_F | TCTTGGAGCGGCCGCA GCCGAGCTGTCCCCC | pTT5 | 8-63 | 36.1 |
| CD234_R | GGTCGGGGGAGGATCC TCATTTCCCGGGAGACAGG |  |  |  |

**Footnotes:** Underline letter of primer sequences indicates it is homologous to vector sequences. The molecular weight of all recombinant proteins includes the expressed protein along with tag. PkDBPa-RII, PkTRAg40.1, and MBP were tagged with hexa-histidine, while PkSBP1 and SPTA1 were tagged with GST, and CD234 was tagged with the Fc region of human Immunoglobulin G1.
