# Supplementary material for: Plasmodium knowlesi tryptophan-rich antigen 40.1 binds human erythrocyte spectrin alpha
Source: PLoS Negl Trop Dis. 2026 Sep 17;20(9):e0014700. doi: 10.1371/journal.pntd.0014700 (PMC13585231; doi:10.1371/journal.pntd.0014700)
Supplement: Supplementary file 3 [file pntd.0014700.s011.docx]

**S2 Table. Peptide sequences of spectrin alpha and hemoglobin subunit beta identified by MALDI-TOF/MS analysis.**

| **Spectrin alpha chain, erythrocytic 1** | |
| --- | --- |
| **Peptide** | **Sequence** |
| **17-27** | K.VLETAEEIQER.R |
| **35-41** | R.YQSFKER.V |
| **61-72** | R.DADDLGKWIMEK.V |
| **259-268** | K.ALSNAANLQR.F |
| **375-389** | R.FSSDFDELSGWMNEK.T |
| **555-563** | K.AIRDGLLAR.R |
| **612-619** | K.DIQNLKSR.V |
| **718-727** | K.GLAEVQNRLR.K |
| **775-785** | R.FEALKEPLATR.K |
| **859-873** | K.MVEEGHFAAEDVASR.V |
| **874-885** | R.VKSLNQNMESLR.A+ Oxidation (M) |
| **982-991** | R.VMALYDFQAR.S+ Oxidation (M) |
| **1017-1032** | K.VEAADHQGIVPAVYVR.R |
| **1034-1045** | R.LAHDEFPMLPQR.R |
| **1034-1046** | R.LAHDEFPMLPQRR.R+ Oxidation (M) |
| **1239-1248** | K.VTILGETAER.L |
| **1265-1277** | K.MELNEAWEDLQGR.T |
| **1420-1434** | R.SDDKSSLDSLEALMK.K |
| **1655-1644** | R.EMLAREDALK.D+ Oxidation (M) |
| **1783-1793** | K.AAVGQEEIQLR.L |
| **1937-1947** | K.ADVVEAWIADK.E |
| **1970-1984** | K.QDTLDASLQSFQQER.L |
| **2005-2015** | K.AIEERYAALLK.R |
| **2017-2028** | R.WEQLLEASAVHR.Q |
| **2080-2094** | R.QLQKDHEDFLASLAR.A |
| **2084-2094** | K.DHEDFLASLAR.A |
| **2282-2294** | K.HFDENLTGRLTHK.E |
| **2291-2297** | R.LTHKEFR.S |
| **2322-2330** | K.FLDAVDPGR.K |
|  |  |
| **Hemoglobin subunit beta** | |
| **Peptide** | **Sequence** |
| **2-9** | M.VHLTPEEK.S |
| **10-18** | K.SAVTALWGK.V |
| **19-31** | K.VNVDEVGGEALGR.L |
| **32-41** | R.LLVVYPWTQR.F |
| **42-60** | R.FFESFGDLSTPDAVMGNPK.V |
| **42-60** | R.FFESFGDLSTPDAVMGNPK.V+ Oxidation (M) |
| **68-83** | K.VLGAFSDGLAHLDNLK.G |
| **97-105** | K.LHVDPENFR.L |
| **122-133** | K.EFTPPVQAAYQK.V |
| **134-147** | K.VVAGVANALAHKYH.- |
